# Therapeutic vaccination of SIV-infected, ART-treated infant rhesus macaques using Ad48/MVA in combination with TLR-7 stimulation

**DOI:** 10.1371/journal.ppat.1008954

**Published:** 2020-10-26

**Authors:** Katherine M. Bricker, Veronica Obregon-Perko, Ferzan Uddin, Brianna Williams, Emilie A. Uffman, Carolina Garrido, Genevieve G. Fouda, Romas Geleziunas, Merlin Robb, Nelson Michael, Dan H. Barouch, Ann Chahroudi

**Affiliations:** 1 Department of Pediatrics, Emory University School of Medicine, Atlanta, GA, United States of America; 2 Duke Human Vaccine Institute, Duke University Medical Center, Durham, NC, United States of America; 3 Departments of Molecular Genetics and Microbiology and Pediatrics, Duke University School of Medicine, Durham, NC, United States of America; 4 Gilead Sciences, Inc., Foster City, CA, United States of America; 5 US Military HIV Research Program, Walter Reed Army Institute of Research, Silver Spring, MD, United States of America; 6 Center for Virology and Vaccine Research, Beth Israel Deaconess Medical Center, Harvard Medical School, Boston, MA, United States of America; 7 Ragon Institute of MGH, MIT, and Harvard, Cambridge, MA, United States of America; 8 Yerkes National Primate Research Center, Emory University, Atlanta, GA, United States of America; 9 Center for Childhood Infections and Vaccines of Children’s Healthcare of Atlanta and Emory University, Atlanta, GA, United States of America; University of Wisconsin, UNITED STATES

## Abstract

Globally, 1.8 million children are living with HIV-1. While antiretroviral therapy (ART) has improved disease outcomes, it does not eliminate the latent HIV-1 reservoir. Interventions to delay or prevent viral rebound in the absence of ART would be highly beneficial for HIV-1-infected children who now must remain on daily ART throughout their lifespan. Here, we evaluated therapeutic Ad48-SIV prime, MVA-SIV boost immunization in combination with the TLR-7 agonist GS-986 in rhesus macaque (RM) infants orally infected with SIV_mac251_ at 4 weeks of age and treated with a triple ART regimen beginning 4 weeks after infection. We hypothesized immunization would enhance SIV-specific T cell responses during ART-mediated suppression of viremia. Compared to controls, vaccinated infants had greater magnitude SIV-specific T cell responses (mean of 3475 vs 69 IFN-*γ* spot forming cells (SFC) per 10^6^ PBMCs, respectively, P = 0.01) with enhanced breadth of epitope recognition and increased CD8^+^ and CD4^+^ T cell polyfunctionality (P = 0.004 and P = 0.005, respectively). Additionally, SIV-specific gp120 antibodies against challenge and vaccine virus strains were significantly elevated following MVA boost (P = 0.02 and P < 0.001, respectively). GS-986 led to expected immune stimulation demonstrated by activation of monocytes and T cells 24 hours post-dose. Despite the vaccine-induced immune responses, levels of SIV DNA in peripheral and lymph node CD4^+^ T cells were not significantly different from controls and a similar time to viral rebound and viral load set point were observed following ART interruption in both groups. We demonstrate infant RMs mount a robust immunological response to this immunization, but vaccination alone was not sufficient to impact viral reservoir size or modulate rebound dynamics following ART release. Our findings hold promise for therapeutic vaccination as a part of a combination cure approach in children and highlight the importance of a pediatric model to evaluate HIV-1 cure interventions in this unique setting of immune development.

## Author summary

While antiretroviral therapy (ART) has improved disease outcome and reduced HIV-1 transmission, it is not a cure, as interruption of ART results in rapid viral rebound due to the persistent latent reservoir. Interventions to induce HIV-1 remission in the absence of ART would be highly beneficial to children living with HIV-1, sparing them from the associated adherence requirements, side effects, and cost of ART. Here, we used our previously established pediatric model of oral SIV infection and ART suppression of viremia in infant rhesus macaques (RMs) to evaluate the safety and efficacy of an Ad48-SIV prime, MVA-SIV boost therapeutic vaccine approach plus TLR-7 stimulation. Our study demonstrates this vaccination strategy is immunogenic in infants; however, unlike previously reported results in adult RMs using a similar approach, vaccination did not result in a difference in the level of CD4^+^ T cell-associated SIV DNA or viral rebound dynamics after ART interruption when compared to control infant RMs. These results highlight the importance of pre-clinical studies using pediatric models and indicate potential HIV-1 cure strategies may differentially impact adults and children.

## Introduction

Globally, 1.8 million children are living with HIV-1 and there are 150,000 new pediatric infections annually [1]. In utero and intrapartum HIV-1 infections have declined with increased understanding of transmission risks and interventions to prevent mother-to-child transmission (MTCT) implemented during pregnancy and delivery. Presently, the majority of new infections occur postnatally through breastmilk transmission [2]. While the global roll-out of antiretroviral therapy (ART) has improved disease outcomes and reduced mortality, ART interruption leads to rapid viral rebound due to reactivation of the persistent latent viral reservoir [3–5]. Therefore, to prevent disease progression and AIDS, HIV-1 infected children must follow lifelong adherence to ART.

HIV-1-infected infants experience rapid progression of disease compared to infected adults. While the median survival for ART-naïve HIV-1-infected adults is 11 years, over 50% of HIV-1-infected children die before the age of two in the absence of ART [6]. Infected infants experience rapid rates of viral production and CD4^+^ T cell turnover, higher peak viremia, a slower decline to viral set point, and set points on the order of 1 log higher than what is observed in HIV-1 infected adults [7]. The mechanisms behind these differences have not been fully elucidated, but are thought to reflect a more immature immune system and inadequate HIV-1-specific immune responses.

While multiple cure strategies have been tested in HIV-1-infected adults, there has been less emphasis on HIV-1-infected children. Only twelve clinical trials focused on targeting HIV reservoirs have been initiated in the pediatric population compared to over eighty in adults [8]. In the first therapeutic vaccine pediatric clinical trial, PEDVAC, DNA vaccination of vertically HIV-1-infected, ART-suppressed children was well tolerated and resulted in a transient increase in the HIV-1-specific cellular immune response [9]. This study was limited by a small sample size, an unboosted vaccine strategy, and the exclusion of an adjuvant. Further trials are necessary to advance this field.

The use of a relevant pediatric animal model may help to provide important safety and efficacy information necessary to bring experimental therapeutics to children. Simian immunodeficiency virus (SIV) infection in the rhesus macaque (RM) has been established as a robust animal model that possesses many similarities to HIV-1 infection including transmission routes, acute infection events, CD4^+^ T cell dynamics, disease progression, and ART-mediated suppression of plasma viral loads [10–12]. This model has been used extensively to inform HIV-1 cure strategies [13–17]; for example, Borducchi et al recently identified a promising therapeutic vaccine regimen that resulted in robust anti-SIV T cell responses leading to a delay in time to viral rebound and reduced set point viremia following ART interruption in vaccinated adult RM compared to ART-only controls [18]. To test hypotheses regarding the viral reservoir, immunity, and remission strategies in a pediatric setting, our laboratory has previously established a model of oral SIV infection in infant RM that effectively simulates postnatal HIV-1 infection through breastfeeding with ART-mediated suppression of viremia [19].

In the present study, we sought to evaluate the immunological and virologic effects of a therapeutic Ad48-SIV prime, MVA-SIV boost immunization strategy in combination with the TLR-7 agonist GS-986 (TV+TLR7) in our model of SIV-infected ART-treated RM infants. We show therapeutic vaccination is safe in a pediatric setting of SIV infection and ART treatment and generates a high-magnitude, broad, and polyfunctional anti-SIV immune response. These results inform our understanding of lentiviral infection and immune responses in a pediatric model and support the inclusion of therapeutic vaccination as a part of a combination cure approach to be tested in children.

## Results

### SIV infection and virologic response to ART

**Fig 1:**
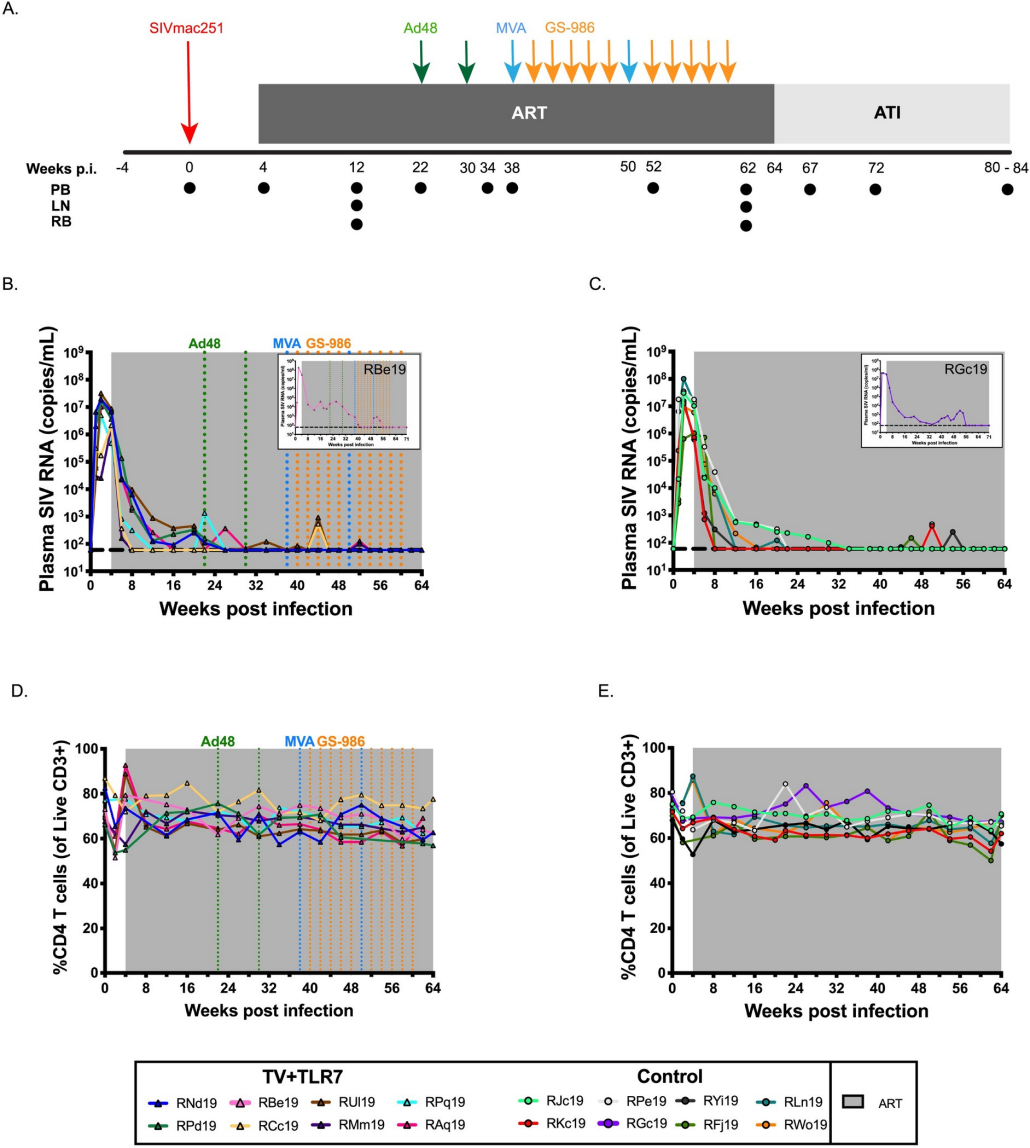
Experimental design and response to ART in SIV-infected infant RMs. (A) Schematic of the study design. Sixteen infant RMs were infected orally with 10^5^ TCID_50_ SIV_mac251_ (day 0), and starting on 4 weeks post infection (p.i.) treated with combination ART (TDF, FTC, DTG) for 15 months. Eight animals received 2 doses of Ad48-SIV_smE543_ *gag-pol-env* (3x10^10^ viral particles, i.m.), 2 doses of MVA-SIV_smE543_ *gag-pol-env* (1x10^8^ PFU, i.m.), and 10 doses of GS-986 (0.3 mg/kg, o.g.), at the timepoints indicated (TV+TLR7). The remaining 8 animals served as ART-treated controls. At 64–71 weeks p.i. RMs underwent analytical treatment interruption (ATI) and all the animals were monitored for 4 to 6 months. PB, RB, and LN biopsies were collected at the indicated timepoints. Longitudinal analysis of plasma SIV RNA levels in (B) TV+TLR7 and (C) control RMs. The shaded area represents the period of ART treatment. The dashed line represents the limit of detection of the assay. Longitudinal analysis of peripheral CD4^+^ T cell frequency in (D) TV+TLR7 and (E) control RMs. The shaded area represents the period of ART treatment.

Sixteen Indian origin RMs (eight males and eight females) were selected for this study. RMs were confirmed negative for the Mamu-B*08 and -B*17 MHC class I alleles associated with natural control of SIV replication. The time course of the experimental design and interventions used are shown in Fig 1A. All RMs were exposed to two consecutive doses of 10^5^ 50% tissue culture infective doses (TCID_50_) SIV_mac251_ by oral administration at approximately 5 weeks of age (range: 3.7 w– 10 w, mean: 5.3 w). Once infection was confirmed by SIV RNA in plasma, no significant difference was noted in pre-ART viral kinetics of infants that required multiple challenges compared to infants successfully infected following first challenge (S1 Fig). As breastfeeding acquisition of HIV-1 is unlikely to be compatible with very early ART initiation, here we started daily ART in all RMs at 4 weeks after SIV infection to approximate this clinical scenario. The ART regimen consisted of two reverse transcriptase inhibitors (tenofovir [TDF] at 5.1 mg/kg of body weight/day and emtricitabine [FTC] at 40 mg/kg/day) and one integrase inhibitor (dolutegravir [DTG] at 2.5 mg/kg/day), co-formulated into a single dose administered once daily by subcutaneous injection for 15 months (as indicated by gray shading in Fig 1A). ART was effective at suppressing SIV RNA in plasma below the limit of detection (LOD) of the assay (60 copies/ml) (Fig 1B and 1C). As also seen in HIV-1-infected children [20–22], time to suppression was variable, ranging from 4 to 52 weeks (median = 15 w) with some infants showing transient blips of viremia (S2 Fig). Two RMs (one each allocated to the vaccine (RBe19) and control (RGc19) groups) sustained significantly higher plasma viral loads on ART than the remainder of the animals (area under the curve [AUC] 1.19x10^5^ ± 5.6x10^4^ vs. 7.58x10 ± 2.8x10^5^; P = 0.0333; Fig 1B and 1C insert); however, both animals did achieve levels of plasma SIV RNA below the LOD after 52 weeks of daily ART. In these two RMs, the experimental design was slightly modified to allow at least 3 months of viral load suppression prior to analytical treatment interruption (ATI).

### Experimental groups and immunization strategy

Prior to the vaccination phase of the experiment, two groups of eight RMs were balanced for sex, age at infection, peak viral load, CD4^+^ T cell frequency at ART initiation, and AUC of pre-ART viremia (Table 1). One group of eight RMs served as ART-only controls. The remaining eight RMs (referred to as TV+TLR7 group) received two immunizations of Ad48 vectors expressing 3x10^10^ viral particles of SIV_smE543_ *gag-pol-env* through the intramuscular (i.m.) route at 22 and 30 weeks post infection (green arrows in Fig 1A). Ad48, like the Ad26-vector used in a prior adult macaque study with a similar immunization strategy [18], is a species D adenovirus associated with gastrointestinal infection and previous studies indicate that both Ad26- and Ad48-vectors induce potent polyfunctional IFN-*γ*^+^, TNF-*α*^+^, and IL-2^+^ T cell responses along with a strong antiviral, pro-inflammatory cytokine and chemokine response [23–25]. Infants were further boosted by two immunizations of MVA vectors expressing 10^8^ plaque forming units of SIV_smE543_ *gag-pol-env* i.m. at 38 and 50 weeks post infection (blue arrows in Fig 1A). Ten doses of the TLR-7 agonist GS-986 were given by orogastric (o.g.) administration at 0.3 mg/kg biweekly at 40, 42, 44, 46, 48, 52, 54, 56, 58, and 60 weeks post infection (orange arrows in Fig 1A). Before administering GS-986 to TV+TLR7 infants, a small dose escalation study was performed in two RMs to monitor for tolerability, safety, and anticipated immune stimulation (S3 Fig), with favorable results found at the 0.3 mg/kg dose. In this dose escalation study and in the 8 TV+TLR7 RMs, we did not observe virus reactivation (measured as on-ART viremia that differed from controls) with GS-986 administration. Overall, ART, Ad48- and MVA-vectors, and GS-986 were well-tolerated, without clinical adverse events throughout the study (S4 Fig).

**Table 1:** Experimental division of SIV-infected, ART-treated infant macaques.

Group	ID	Sex	A01 Status	Age at infection, weeks	Virus	CD4 Freq, baseline	CD4 Freq, ART initiation	Peak PVL, pre-ART	AUC PVL, pre-ART
**ART-only controls**	RJc19	M	+	4.4	SIV_mac251_	73.5%	68.5%	3.05E+07	5.61E+07
	RKc19	F	+	4.1	SIV _mac251_	70.6%	67.1%	1.27E+07	2.00E+07
	RPe19	M	-	4.3	SIV _mac251_	73.0%	65.1%	3.51E+07	8.81E+07
	RGc19	M	-	9.6	SIV_mac239_	76.3%	68.3%	4.32E+07	1.39E+08
	RYi19	F	-	3.9	SIV _mac251_	50.6%	53.8%	1.56E+07	2.40E+07
	RFj19	F	-	5.4	SIV _mac251_	69.6%	n/a	1.03E+06	1.99E+06
	RLn19	M	-	4.4	SIV _mac251_	72.7%	62.7%	1.01E+08	1.71E+08
	RWo19	F	-	3.7	SIV _mac251_	74.5%	62.4%	9.84E+06	2.07E+07
**TV+TLR7**^a^	RNd19	M	-	5.4	SIV _mac251_	65.2%	56.8%	1.98E+07	4.32E+07
	RPd19	F	-	5.3	SIV _mac251_	81.9%	74.1%	1.78E+07	3.24E+07
	RBe19	F	-	5	SIV _mac251_	80.1%	80.2%	1.98E+08	3.30E+08
	RCc19	M	+	10	SIV_mac239_	85.7%	70.4%	2.16E+06	2.73E+06
	RUl19	M	-	4.9	SIV _mac251_	67.5%	62.7%	3.20E+07	5.68E+07
	RMm19	M	-	5.9	SIV _mac251_	70.5%	62.9%	1.85E+06	1.92E+06
	RPq19	F	+	3.3	SIV _mac251_	76.9%	73.7%	5.01E+06	8.07E+06
	RAq19	M	+	4.9	SIV _mac251_	69.7%	64.1%	1.03E+07	2.31E+07

^a^TV+TLR7, therapeutic vaccination with Ad48/MVA+GS-986.

### Cellular immune response to therapeutic vaccination

**Fig 2:**
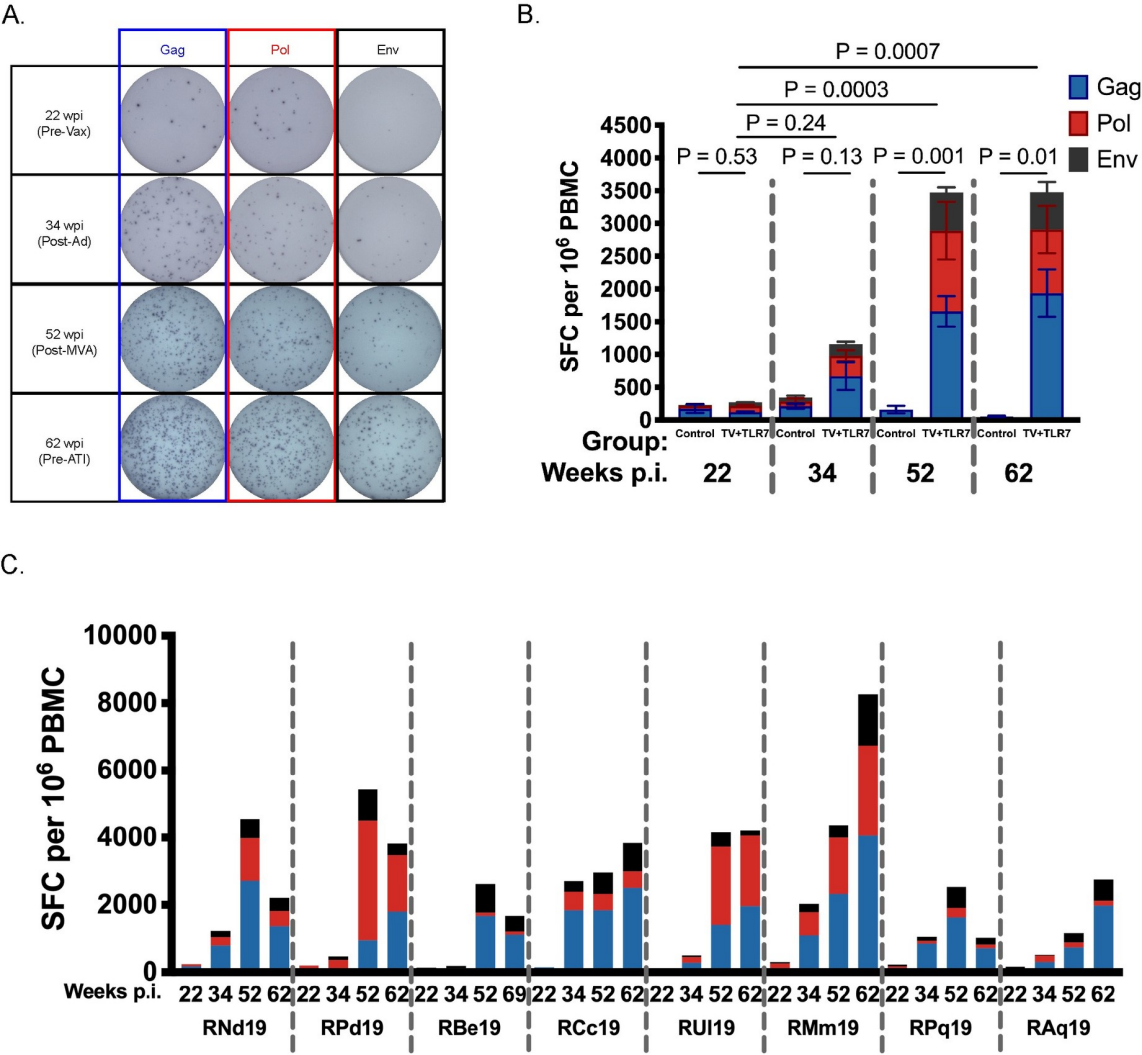
Cellular response measured by ELISPOT to therapeutic vaccination in SIV-infected, ART-treated infant RMs. (A) Representative images of spot forming cells (SFC) in response to Gag, Pol, and Env at timepoints indicated above. Wpi, weeks post infection. (B) IFN-*γ* ELISPOT responses to Gag, Pol, and Env peptide pools from SIV_mac239_ were measured at week 22 prior to vaccination, week 34 after priming with two doses of Ad48, week 52 after boosting with two doses of MVA, and week 62 prior to analytical treatment interruption in control RMs (n = 4) and TV+TLR7 (n = 8) RMs. Bars represent mean ± SEM. Statistical analysis was performed to compare with week 22 using the non-parametric Friedman test with Dunn's multiple comparison test to correct for multiple comparisons. (C) Individual IFN-*γ* ELISPOT responses of TV+TLR7 RMs.

To evaluate the immunogenicity of the Ad48/MVA vaccine approach we performed IFN-*γ* ELISPOT assays at week 22 prior to vaccination, week 34 after priming with Ad48, week 52 after boosting with MVA, and 2 weeks prior to ATI. Representative data showing the increase in Gag-, Pol-, and Env-specific spot forming cells over this time course are shown in Fig 2A. Vaccinated RMs demonstrated a robust increase in the magnitude of Gag-, Pol-, and Env-specific cellular immune responses against SIV_mac239_ peptides after Ad48 prime that were boosted following MVA (P = 0.0003, Fig 2B, n = 8). In ART-only controls, the SIV-specific immune response remained low throughout ART treatment (Fig 2B, n = 4) with a decline in spot forming cells observed during ART (S5 Fig). Variability in the magnitude of responses in the TV+TLR7 group was observed (Fig 2C) and, interestingly, 3 RMs (RCc19, RMm19, RAq19) had evidence of increasing SIV-specific immunity from 2 to 10 weeks after the final MVA dose during the period of GS-986 administration.

**Fig 3:**
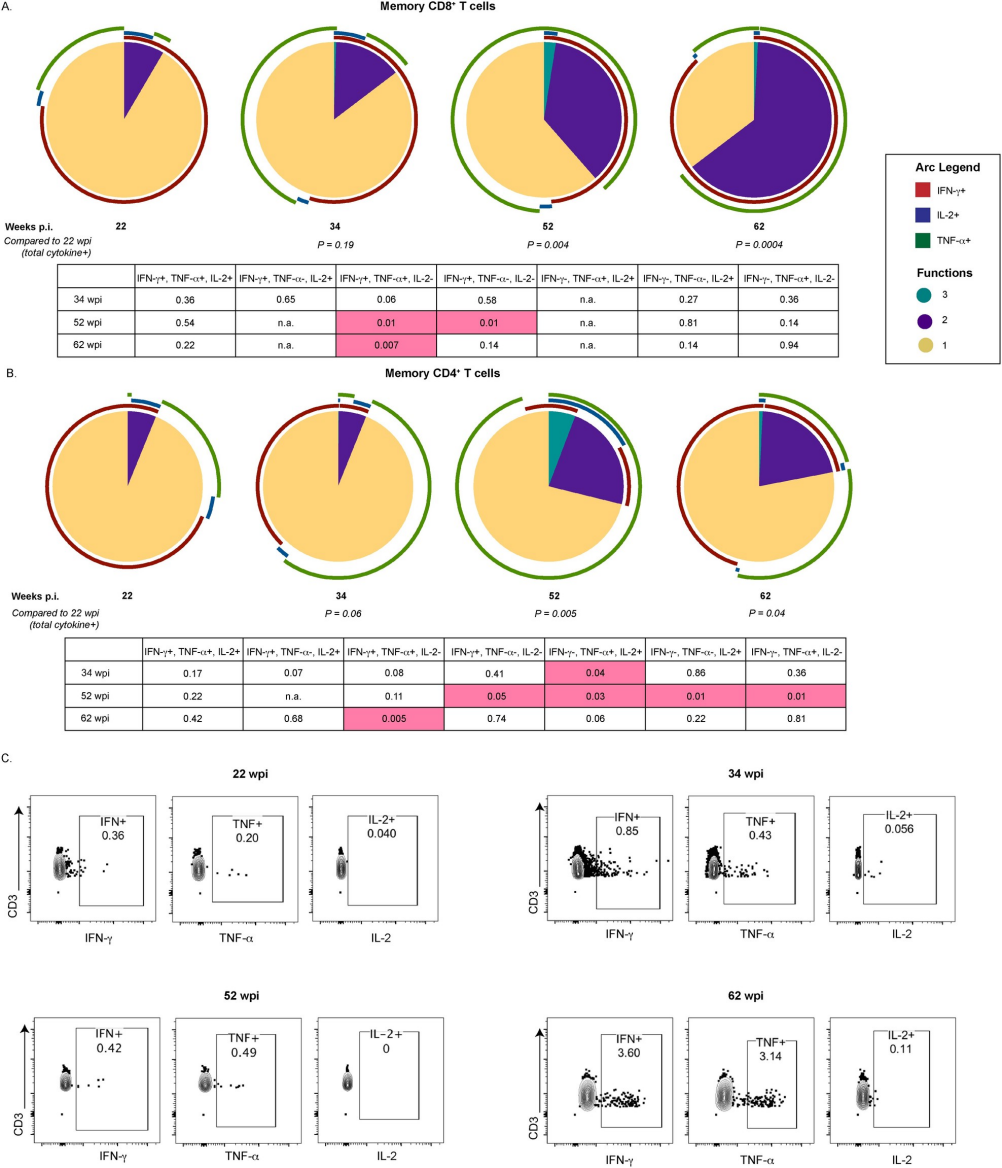
Immunological response measured by ICS to therapeutic vaccination in SIV-infected, ART-treated infant RMs. Pie charts depicting the ability of (A) memory CD8^+^ and (B) memory CD4^+^ T cells isolated from TV+TLR7 RMs to produce IFN-γ, IL-2 and/or TNF-*α* in response to stimulation with SIV_mac239_ Gag peptide pool at 22 (n = 5), 34 (n = 6), 52 (n = 4), and 62 (n = 7) weeks post infection (wpi). Total cytokine positive cells were compared to week 22 through permutation test. Cytokine positive subsets were compared to week 22 using a Wilcoxon Rank Sum Test, table representing P values is shown. (C) Representative flow plot of IFN-*γ*, TNF-*α*, and IL-2 expression in CD95^+^CD8^+^ T cells.

When cell availability permitted, SIV-specific T cell responses were further interrogated through intracellular cytokine staining (ICS) following stimulation with SIV Gag peptide pools at the timepoints described above. Boolean gating was used to examine Gag-specific T cells for polyfunctional responses associated with enhanced HIV-1 control [26, 27]. The frequency of memory CD8^+^ T cells (gated as CD95^+^) with 2 or more functions significantly increased after MVA boost (week 52, P = 0.004) and remained significant prior to ATI (week 62, P = 0.0004) compared to the pre-vaccination timepoint (Fig 3A). The largest increase was observed in cells double positive for IFN-*γ* and TNF-*α* (P = 0.007). In memory CD4^+^ T cells the most dramatic increase in Gag-specific cells was observed after MVA boost (P = 0.005) with a significant increase observed in TNF-*α* and IL-2 double positive memory CD4^+^ T cells (P = 0.03). The frequency of Gag-specific cytokine positive memory CD4^+^ T cells remained significant prior to ATI (P = 0.04) with the IFN-*γ* and TNF-*α* double positive memory CD4^+^ T cells subset significantly elevated when compared to week 22 (P = 0.005) (Fig 3B). A representative flow plot of memory CD8^+^ T cells expressing IFN-*γ*, TNF-*α*, and IL-2 at each described timepoint is shown in Fig 3C.

### Breadth of SIV-specific immune responses

**Fig 4:**
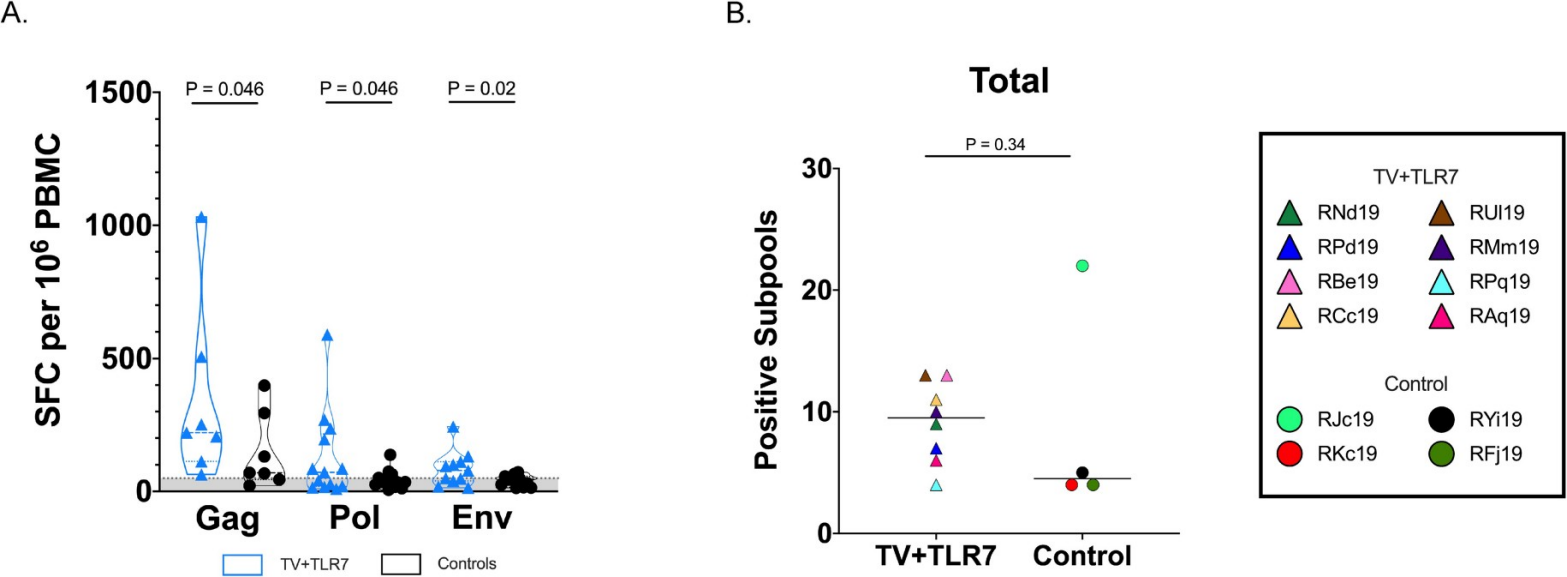
Cellular breadth of immunological response following analytical treatment interruption (ATI) measured by ELISPOT to therapeutic vaccination in SIV-infected, ART-treated infant RMs. (A) IFN-*γ* ELISPOT responses to 10-mer peptide subpools spanning the Gag, Pol, and Env proteins from SIV_mac239_ following ATI in TV+TLR7 RMs and control RMs. Bars represent median ± quartiles and the gray shading bordered by the horizontal dashed line represents the limit of detection. Statistical analysis was performed using Wilcoxon matched-pairs signed rank tests. SFC, spot forming cells. (B) Cellular immune breadth in TV+TLR7 RMs and control RMs as measured by total positive subpools of 10 peptides spanning the SIV_mac239_ Gag, Pol, and Env proteins following ATI. Black bar represents median. Groups were compared using a two-sided Mann-Whitney test (P < 0.05 was considered significant).

To estimate cellular breadth, 10-mer peptides were divided into subpools of 20 peptides spanning the entire Gag (7 subpools), Pol (13 subpools), or Env (11 subpools) proteins from SIV_mac239_. Vaccinated RMs demonstrated a significantly higher magnitude of cellular responses to Gag, Pol, and Env viral subpools when compared to ART-only controls (P = 0.046, 0.046, and 0.02, respectively; Fig 4A). Responses varied by RM and subpool (S6A Fig). Although not significant, the total number of positive subpools was higher in TV+TLR7 RMs than ART-only controls (median = 9.5 and 4.5, respectively) (Fig 4B). This finding held true for subpools for each viral protein (S6B Fig). These analyses were performed using a subset of control RMs with sufficient cells and, surprisingly, one control RM (RJc19) had the highest observed breadth overall. If RJc19 is excluded from comparison in Fig 4B, the results do reach significance (P = 0.04) with a greater number of positive subpools in the TV+TLR7 group.

## Humoral immune response to therapeutic vaccination

**Fig 5:**
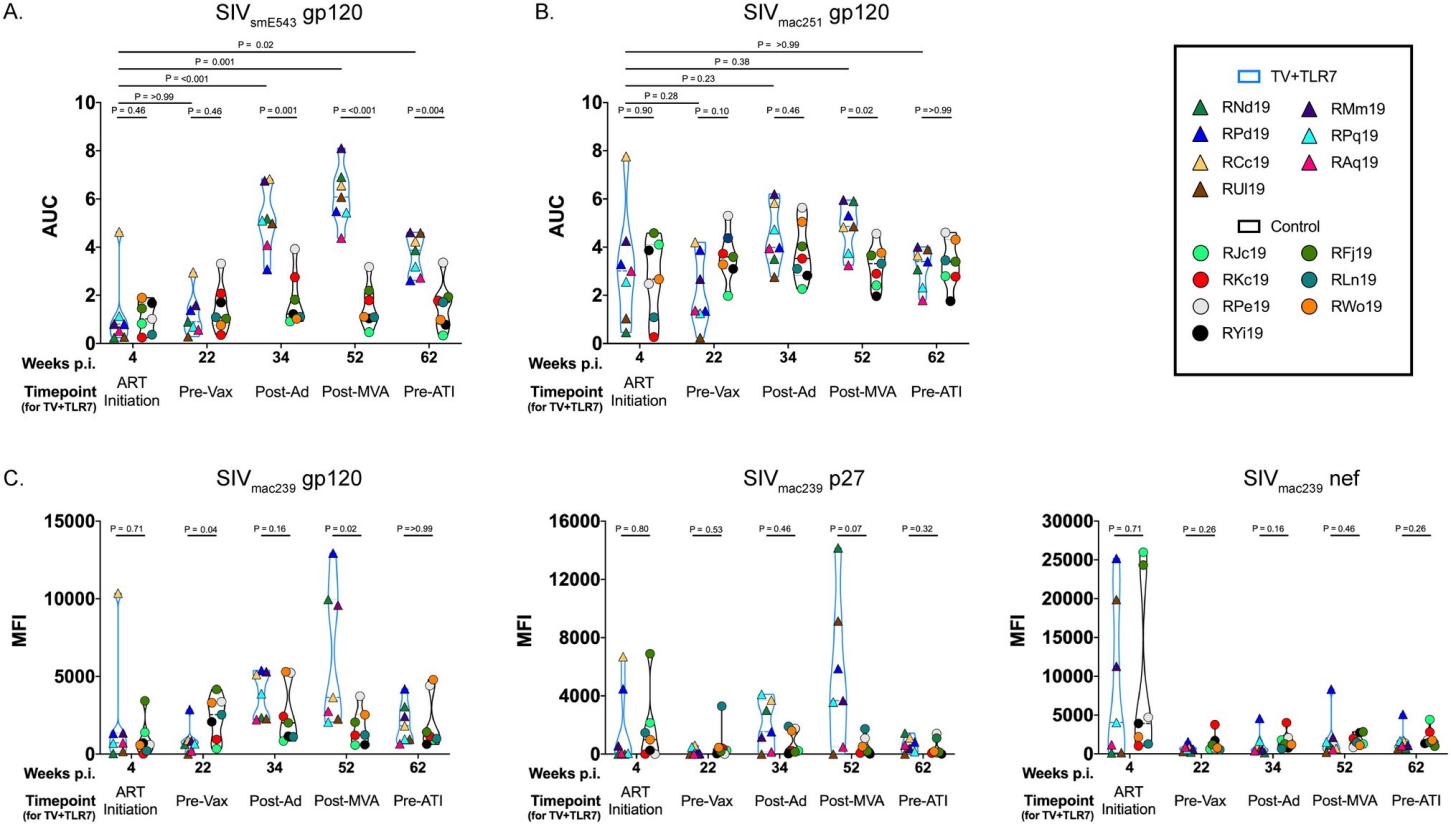
Humoral response measured by ELISA and BAMA to therapeutic vaccination in SIV-infected, ART-treated infant RMs. SIV-specific antibodies directed against gp120 from (A) vaccine strain, SIV_smE543_, and (B) challenge strain, SIV_mac251_, were quantified by binding ELISA area under the curve (AUC) at week 4 prior to ART initiation, week 22 prior to vaccination, week 34 after priming with two doses of Ad48, week 52 after boosting with two doses of MVA, and week 62 prior to analytical treatment interruption (ATI) in TV+TLR7 RMs and equivalent time points for controls. (C) SIV-specific antibodies directed against SIV_mac239_ gp120, p27, and Nef were measured by Binding Antibody Multiplex Assay (BAMA) at same time points described above. Experimental groups were compared using a two-sided Mann-Whitney test and timepoints within groups were compared using the non-parametric Friedman test with Dunn's multiple comparison test to correct for multiple comparisons (P < 0.05 was considered significant).

To further explore the immunological impact of TV+TLR7 we performed ELISAs at week 4 after infection and prior to ART initiation, week 22 prior to vaccination, week 34 after priming with Ad48, week 52 after boosting with MVA, and 2 weeks prior to ATI. The presence of SIV-specific gp120 antibodies was assessed against the vaccine strain virus, SIV_smE543_, and challenge strain virus, SIV_mac251_ (Fig 5A and 5B, respectively). Vaccinated RMs demonstrated a significant increase in binding antibodies against SIV_smE543_ gp120 following Ad26-prime (P < 0.001 compared to week 4 in the TV+TLR7 group and P = 0.001 compared to controls at week 34). The gp120 reactivity peaked at week 52 following the MVA boost (P = 0.001 compared to week 4 in the TV+TLR7 group and P < 0.001 compared to controls at week 52) and remained significantly higher in TV+TLR7 RMs versus controls at week 62 (2 weeks prior to ATI; P = 0.004; Fig 5A). Antibodies directed against SIV_mac251_ gp120 also peaked at week 52 in TV+TLR7 RMs (P = 0.02 compared to controls at the same timepoint), but declined to a level similar to control RMs by week 62 (Fig 5B). Two RMs (RBe19 in the TV+TLR7 group and RGc19 in the control group) were excluded from these analyses since they remained viremic on ART for a prolonged period (Fig 1C insert) and antibody responses could not be reliably attributed to antigen exposure through vaccination.

To evaluate the heterogeneity of the antibody response, we additionally performed a Binding Antibody Multiplex Assay (BAMA) at the above described timepoints against SIV_mac239_ gp120, p27, and Nef. We did not find an increase in SIV_mac239_-specific antibodies in TV+TLR7 RMs following immunization compared to week 4, although at week 52 after MVA boost gp120-specific antibodies were significantly higher (P = 0.02) and p27-specific antibodies trended higher (P = 0.07) compared to controls (Fig 5C). Binding antibodies against the Nef protein, which was not included in the vaccine insert, were elevated in some RMs from both groups prior to ART initiation but then remained low throughout the subsequent timepoints.

### Cellular kinetics following GS-986

**Fig 6:**
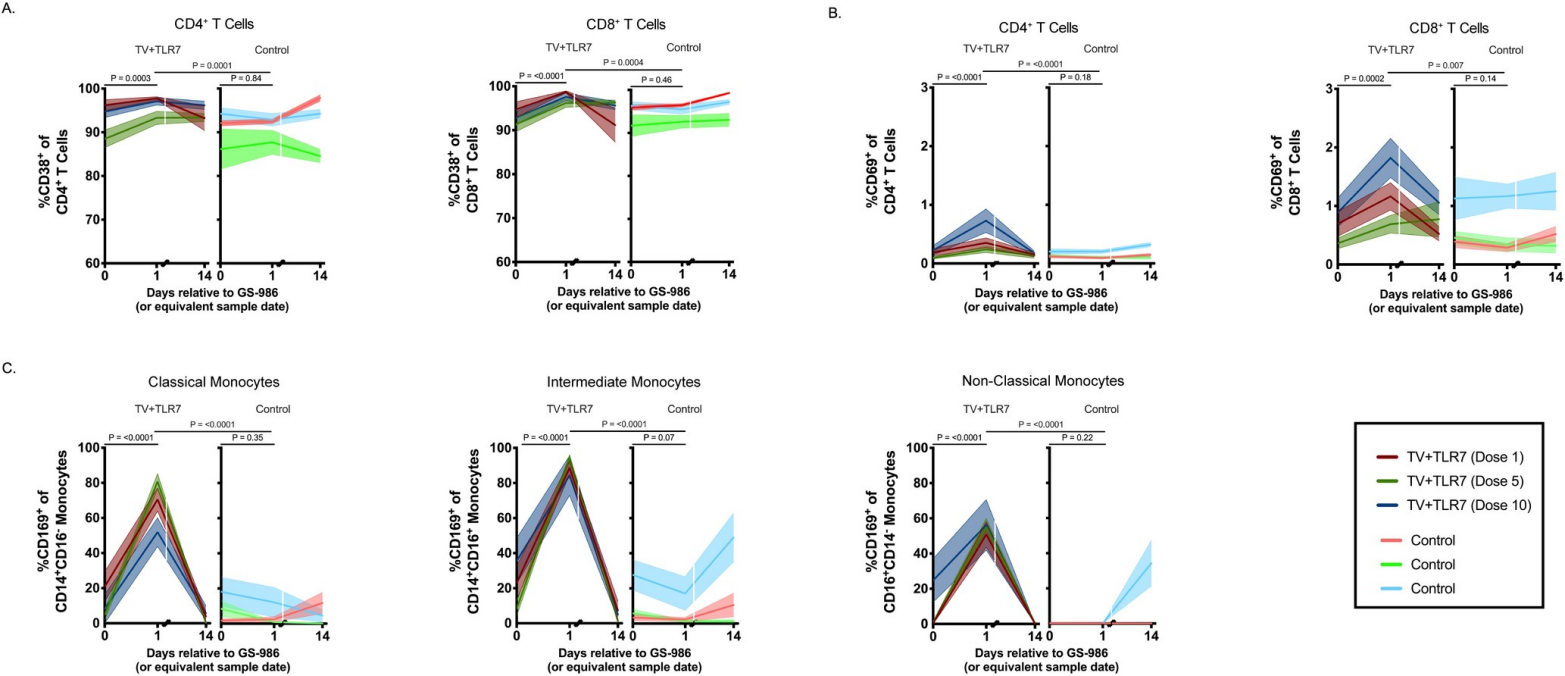
Immunological response to repeated oral GS-986 administration in SIV-infected, ART-treated infant RMs. Frequency of (A) CD38^+^ and (B) CD69^+^ peripheral CD4^+^ and CD8^+^ T cells immediately prior to, 1 day post, and 14 days post oral GS-986 dose 1, 5, and 10 in TV+TLR7 or equivalent sample day in control RMs. (C) Frequency of CD169^+^ classical, intermediate, and nonclassical monocytes in TV+TLR7 and control RMs. Dose number is indicated by color. Bars represent mean ± SEM. Statistical analysis was performed using a Wilcoxon matched-pairs signed rank test.

To investigate the immunological response to repeated oral GS-986 administration, blood was collected pre-, 24 hours post-, and 2 weeks post-GS-986 doses 1, 5, and 10. These timepoints were selected to allow longitudinal evaluation while remaining within limited blood availability constraints due to the size of the infants. Whole blood flow cytometry was performed to evaluate peripheral immune cell activation and kinetics. The frequency of CD38 expression on CD4^+^ and CD8^+^ T cells was high at baseline with a mean of 93% for each in TV+TLR7 RMs, then transiently increased to a mean of 96% and 97% at the 24-hour post-dose timepoint in TV+TLR7 macaques (P = 0.0003 and P < 0.0001, respectively, Fig 6A and S7A Fig). CD38 expression on both CD4^+^ and CD8^+^ T cells was significantly higher in TV+TLR7 than control RMs at the 24-hour timepoint (P = 0.0001 and P = 0.0004, respectively) and no significant increase from baseline was observed in control RMs. CD38 expression on both CD4^+^ and CD8^+^ T cells returned to baseline levels by 2 weeks post GS-986 in the TV+TLR7 group. Similarly, CD69 expression significantly increased from a mean of 0.17% to a mean of 0.44% in CD4^+^ T cells and from a mean of 0.65% to 1.2% in CD8^+^ T cells (P < 0.0001 and P = 0.0002, respectively, Fig 6B and S7B Fig). For both CD4^+^ and CD8^+^ T cells, CD69 expression was significantly higher in TV+TLR7 RMs than control RMs measured at the 24-hour timepoint (P < 0.0001 and P = 0.007, respectively). Overall, responses were similar after doses 1, 5, or 10.

CD169, or Siglec-1, is upregulated on monocytes and macrophages following stimulation of TLR-7 or exposure to type 1 interferons produced by plasmacytoid dendritic cells [28]. CD169^+^ monocytes then travel to the tissue, differentiate, and act to promote adaptive immune responses to pathogens through antigen presentation and secretion of cytokines. For this reason, we sought to evaluate the expression of CD169 on monocytes in the periphery to further validate the immune stimulatory effect of oral GS-986 in RM infants. For this analysis, monocytes were divided into classical (CD14^+^CD16^-^), intermediate (CD16^+^CD14^+^), and non-classical (CD16^+^CD14^-^) subsets. The frequency of CD169 expression on classical, intermediate, and non-classical monocyte subsets was low at baseline with a mean of 11.4, 22.4, and 8.7%, respectively, then transiently increased to a mean of 68, 89, and 54% (P < 0.0001 for each comparison) at the 24-hour post GS-986 timepoint in TV+TLR7 macaques (Figs 6C and S7C). In control RMs, CD169 expression remained mostly stable over the same time period, although some non-statistically significant fluctuations were observed. As with T cell activation, monocyte stimulation was similar after doses 1, 5, or 10 in the TV+TLR7 group.

### TV+TLR7 impact on persistent SIV

**Fig 7:**
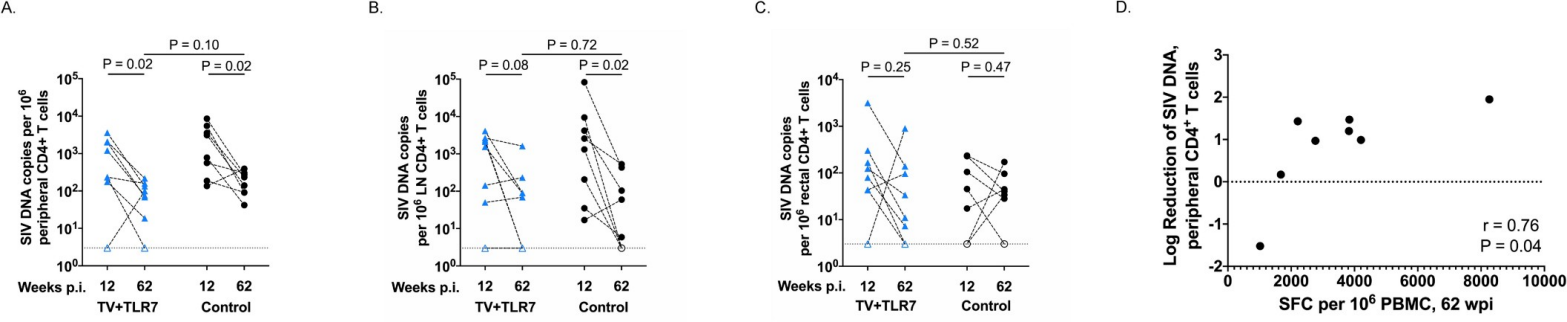
Impact of therapeutic vaccination and oral TLR-7 stimulation on SIV DNA persistence in CD4^+^ T cells of SIV-infected, ART-treated infant RMs. Comparison of frequency of estimated SIV DNA levels in (A) peripheral, (B) LN, and (C) rectal CD4^+^ T cells before (12 weeks post infection, wpi) and after (62 wpi) therapeutic vaccine regimen in TV+TLR7 and control RMs as determined by PCR. Dashed line represents the limit of detection and open symbols represent values below the limit of detection. Statistical analysis was performed using a two-sided Wilcoxon rank-sum tests (P < 0.05 was considered significant). (D) Association between log reduction of SIV DNA in peripheral CD4^+^ and LN CD4^+^ T cells from pre-vaccination to post-vaccination and magnitude of SIV-specific T cells at week 62 prior to ART interruption measured by IFN-*γ* ELISPOT in TV+TLR7 RMs. Two-sided Spearman rank correlation test was used to determine statistical significance. *R* value indicates correlation coefficient.

To evaluate the impact of the therapeutic vaccine regimen on SIV persistence on ART, the level of total cell-associated SIV DNA was measured longitudinally in CD4^+^ T cells isolated from whole blood, lymph nodes, and colorectal mucosa. In TV+TLR7 RMs, we observed a significant reduction of 0.8 log in SIV DNA in peripheral CD4^+^ T cells from week 12 prior to vaccine regimen and week 62 after completion of the vaccine regimen (P = 0.02, Fig 7A). A trend towards reduced levels of SIV DNA in CD4^+^ T cells isolated from lymph nodes was also observed over the same time course (P = 0.08, Fig 7B). We note, however, that neither the frequency of CD4^+^ T cells with SIV DNA prior to ATI or log change over the observed time course significantly differed in TV+TLR7 infants compared to ART-only controls. Levels of SIV DNA in rectal CD4^+^ T cells in TV+TLR7 and control RMs remained stable from week 12 to week 62 (Fig 7C). Despite these similar findings between groups, we demonstrate that the log reduction in CD4^+^ T cell-associated SIV DNA in the periphery was significantly positively correlated with the magnitude of the SIV-specific T cell response in TV+TLR7 infants prior to ATI at week 62 (r = 0.76, P = 0.04, Fig 7D). The significance of this correlation was lost when values for ART-only control RMs were added to the analysis (r = 0.37, P = 0.24) or when compared in ART-only controls alone (r = -0.2, P = 0.92).

### Viral rebound dynamics

**Fig 8:**
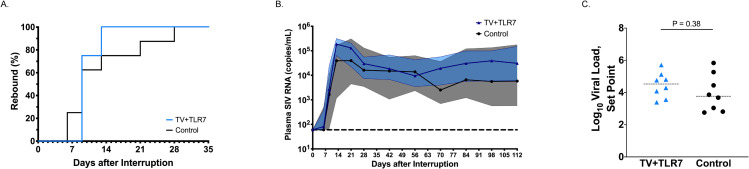
Influence of TV+TLR7 on time to rebound and post-rebound viremia following analytical treatment interruption (ATI) in SIV-infected, ART-treated infant RMs. (A) Comparison of time to viral rebound in TV+TLR7 and control RMs depicted by Kaplan-Meier curves. Survival curves for groups were compared through Log-rank (Mantel-Cox) test. (B) Median longitudinal plasma SIV RNA levels in TV+TLR7 and control RMs following ATI. The solid line represents the median, the shaded area represents interquartile range, and the horizontal dashed line represents the limit of detection of the assay. (C) Set point viremia following 16 weeks of ATI of TV+TLR7 and control RMs determined by the mean of the final three viral load measurements. Dashed bars represent median. Groups were compared using a two-sided Mann-Whitney test (P < 0.05 was considered significant).

To evaluate the impact of TV+TLR7 on the replication competent viral reservoir that contributes to viral rebound upon discontinuation of ART, RMs underwent ATI following 60–67 weeks of daily ART. Plasma viral loads were monitored at days 6, 9, 13, 21, and 28 after ART interruption to determine time to viral rebound and RMs were followed for 16 weeks off ART to trend viral set points. The time to viral rebound was defined as the first of two consecutive timepoints with viremia greater than 500 copies/mL. All RMs rebounded by 28 days post-ATI with no significant difference in time to rebound between TV+TLR7 and control groups (median = 9 days for both groups; P = 0.6) (Fig 8A). Similarly, no differences were detected in post-rebound AUC or set point viremia (defined as the mean of the final three plasma viral loads prior to experimental end point) in animals that received the vaccine regimen compared to ART-only controls (median = 4.37 and 3.66 log SIV RNA copies/ml of plasma, respectively, P = 0.38) (Fig 8B and 8C, S8 Fig).

### Strong and weak responders within the TV+TLR7 group

**Fig 9:**
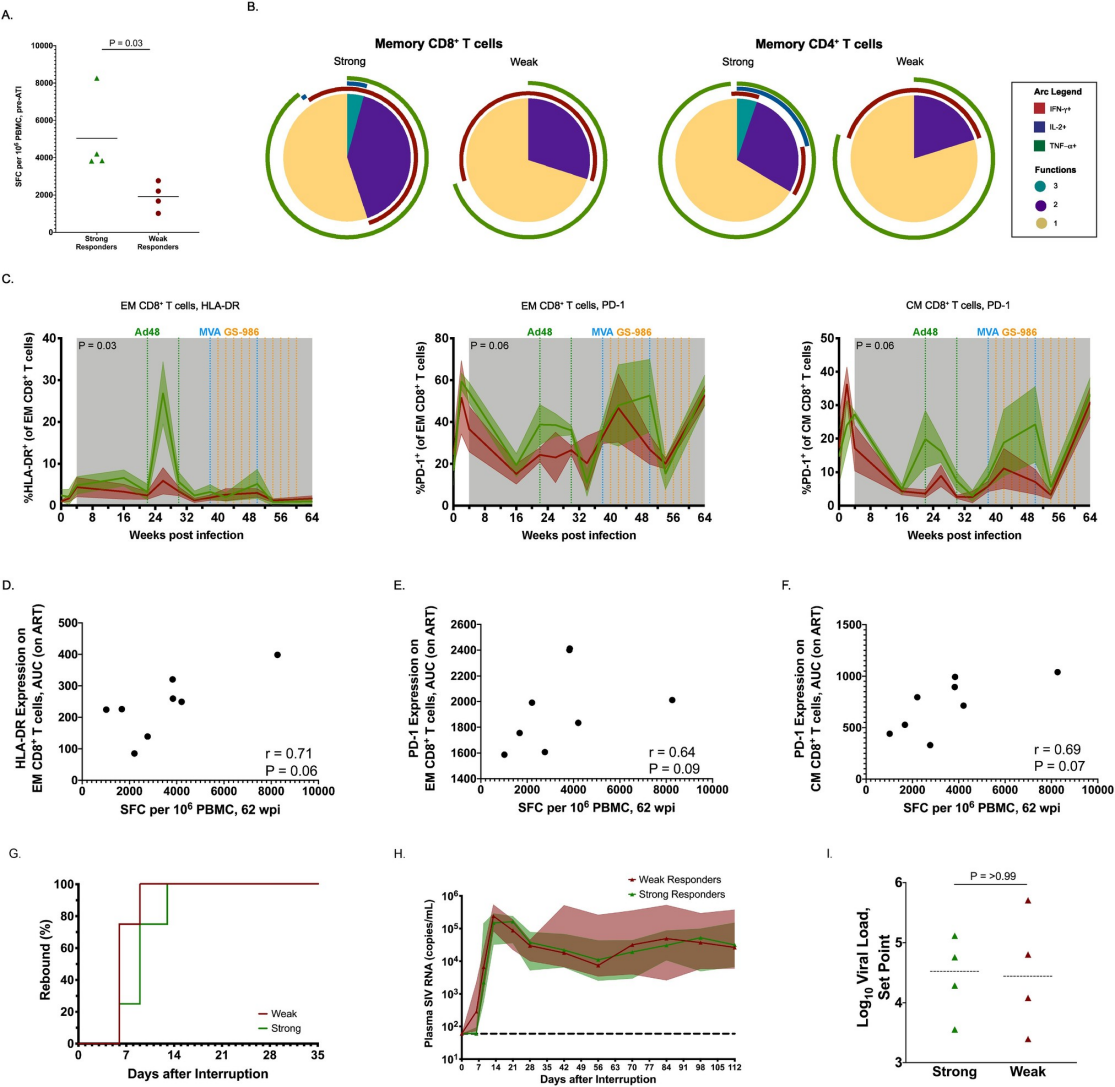
Comparison of strong and weak cellular vaccine response within TV+TLR7 infant RMs. (A) Magnitude of anti-SIV cellular immunity measured by IFN-*γ* ELISPOT prior to ATI at 62 weeks post infection of strong and weak responders. Dashed bars represent median. Groups were compared using a two-sided Mann-Whitney test (P < 0.05 was considered significant). (B) Pie charts depicting the ability of memory CD8^+^ and CD4^+^ T cells isolated from strong and weak TV+TLR7 responders to produce IFN-γ, IL-2 and/or TNF-*α* in response to stimulation with SIVmac239 Gag peptide pool at 52 weeks post infection (n = 4). (C) Longitudinal analysis of HLA-DR and PD-1 expression on effector memory (EM) and central memory (CM) CD8^+^ T cells in strong and weak responders within TV+TLR7 RMs (n = 8). The shaded area represents the period of ART treatment. AUC on ART was compared between groups using a two-sided Mann-Whitney test. (P < 0.05 was considered significant) Association between (D) HLA-DR expression on EM, (E) PD-1 expression on CM, and (F) PD-1 expression on EM CD8^+^ T cells during ART represented by AUC and magnitude of SIV-specific T cells following end of vaccine regimen prior to ATI measured by ELISPOT in TV+TLR7 RMs. Two-sided Spearman rank correlation test was used to determine statistical significance. *R* value indicates correlation coefficient. (G) Comparison of time to viral rebound in strong and weak TV+TLR7 responder RMs depicted by Kaplan-Meier curves. (H) Median longitudinal plasma SIV RNA levels between strong and weak responders following ATI. The solid line represents the median, the shaded area represents interquartile range, and the horizontal dashed line represents the limit of detection of the assay. (I) Set point viremia following 16 weeks of ATI of strong and weak TV+TLR7 responders determined by the mean of the final three viral load measurements. Dashed bars represent median. Groups were compared using a two-sided Mann-Whitney test (P < 0.05 was considered significant).

As mentioned, there was variability in the immune response induced by TV+TLR7 in RM infants. We explored potential correlates of this variability by dividing the TV+TLR7 infants into “strong” (RPd19, RCc19, RUl19, & RMm19) and “weak” (RNd19, RBe19, RPq19, RAq19) cellular responders based on the magnitude of their anti-SIV T cell responses at week 62 prior to ATI, with significantly more IFN-*γ*-producing T cells in the four best responders compared to the four poorest responders (mean = 5033 vs. 1917 SFC per 10^6^ PBMC, P = 0.03, Fig 9A). In the subset of 5 TV+TLR7 RMs for which ICS data is available, CD8^+^ and CD4^+^ T cells triple positive for IFN-*γ*, IL-2, and TNF-*α* were only observed in the strong responders as defined above following the final MVA boost. Moreover, memory CD4^+^ T cells with 2 or 3 functions were significantly increased in strong vs. weak responders (P = 0.03, Fig 9B).

We then sought to explore immunological or virologic factors that could explain these differential vaccine responses. A distinct immunological activation signature was present in memory CD8^+^ T cells between strong and weak vaccine responders (Fig 9C). Longitudinal HLA-DR expression, measured as AUC, on effector memory CD8^+^ T cells was significantly higher in strong vaccine responders compared to weak responders (P = 0.03). A correlation was observed between HLA-DR expression on effector memory CD8^+^ T cells during ART and the magnitude of the anti-SIV T cell response at week 62 (r = 0.71, P = 0.06), (Fig 9D). Although not significant, area under-the-curve of longitudinal PD-1 expression in the strong vaccine responders in both effector and central memory CD8^+^ T cells also tended to be higher than weak vaccine responders (P = 0.09 and 0.07, respectively). Furthermore, PD-1 expression was positively associated with SIV-specific T cell responses at week 62 (Fig 9E and 9F). Despite these described differences, there were no significant differences between strong and weak responders in the levels of CD4^+^ T cell-associated SIV DNA prior to ATI or in the log reduction in cell-associated SIV DNA after vaccination (S9 Fig). Additionally, time to viral rebound, post-rebound viremia AUC, and viral set point were similar between the strong and weak responder groups (Fig 9G–9I). Finally, across all animals in both the TV+TLR7 and ART-only control groups no associations between any of the measured immunological parameters and viral rebound kinetics were identified.

## Discussion

This study provides the first insight into the use of a viral vectored therapeutic vaccine approach as a potential cure intervention in a pediatric model of oral HIV-1 infection and long-term ART suppression. We demonstrate safety and immunogenicity of an Ad48 prime, MVA boost immunization in combination with repeated oral TLR-7 stimulant GS-986 in SIV-infected, ART-treated infant RMs.

HIV/SIV-specific CD8^+^ T cell responses are low during ART [29] and prior to the results presented here, it was unclear how much they could be boosted through therapeutic vaccination of infants. As ART was started 4 weeks after SIV infection in this study, a de novo CD8^+^ T cell response to the infecting viral strain was likely elicited with sufficient time for viral escape mutations to develop, which could potentially impair the vaccine response. Based on the strong cellular immune response evident in vaccinated animals following completion of the Ad48/MVA regimen that was significantly increased above their pre-vaccine levels and in comparison to unvaccinated controls, we conclude that this vaccine regimen was immunogenic. The vaccine strain SIV_smE543_ shares sequence identity to SIV_mac239_ of 91, 93, and 85%, for Gag, Pol, or Env, respectively, so it is possible we did not fully capture the extent of vaccine elicited responses, especially to Env, with the SIVmac239 peptide pools. However, SIV_mac239_ shares sequence identity of 97% to the infecting virus (SIV_mac251_) which we anticipated would be the most relevant to target.

In addition to enhanced cellular immunity we have demonstrated that therapeutic vaccination with TLR-7 stimulation also enhances the humoral immune response. Binding antibodies against gp120 from SIV_smE543_, SIV_mac251_, and SIV_mac239_ peaked at week 52 following the MVA boost. Antibodies directed against SIV_mac239_ p27 were also significantly higher than those measured in control RMs at the week 52 timepoint. A decline in gp120 reactivity in TV+TLR7 RMs was observed following the end of the vaccine regimen and prior to ATI such that immunized animals did not differ from controls in response to gp120 from the challenge (and therefore rebounding) strain, although immunized animals did retain a higher response to the vaccine strain gp120. Incorporating a protein boost to this vaccination approach may maintain humoral immune responses for a longer period and therefore have a greater impact on viral rebound kinetics. A more in-depth investigation of the impact of therapeutic vaccination on the humoral immune response, including functional assays, will likely provide additional insight into the role of vaccine induced antibodies to control viral rebound after ART interruption.

Repeated oral TLR-7 stimulation by GS-986 resulted in transient immune activation, as previously demonstrated in studies with adult RMs [18, 30]. CD38 and CD69 expression was significantly elevated at 24 hours post-doses 1, 5, and 10 when compared to baseline in CD4^+^ and CD8^+^ T cells. Monocyte activation was also evident at this timepoint, with CD169 expression significantly increased on classical, intermediate, and non-classical monocytes. This study is the first reported use of an oral TLR-7 agonist in infants and provides critical safety and immunogenicity data for future pediatric trials. In our hands, GS-986 was not an effective latency reversal agent. We did observe several blips of viremia in treated RMs, but similar viral blips were also seen in the control group around the same timepoints indicating that this transient viremia was likely unrelated to GS-986 treatment (Fig 1B and 1C). Our findings conflict with Lim *et al*. [30], but are consistent with reports by Del Prete *et al*, and Borducchi *et al*, and Bekerman *et al*. [18, 31–33]. Based on the safe induction of innate immune activation we observed in infants, TLR-7 agonists remain promising for use as vaccine adjuvants or perhaps could be explored in combination with other agents to stimulate virus reactivation from latency.

The impact of therapeutic vaccination on the viral reservoir prior to ATI was estimated through quantification of total SIV DNA in peripheral and lymph node CD4^+^ T cells. No significant difference was found in the frequency of SIV^+^ CD4^+^ T cells prior to ATI or the reduction of SIV^+^ CD4^+^ T cells during ART regimen between vaccinated and ART-only control RMs. However, an association was observed between the magnitude of the vaccine response prior to ATI and the reduction in the frequency of SIV^+^ CD4^+^ T cells in TV+TLR7 infants that was not present in control RMs. These results imply that in the immunized infants, the observed decline in infected cells may be attributed, in part, to the vaccine-induced immune response. We acknowledge measurement of total SIV DNA represents an overestimate of the viral reservoir that includes cells containing defective virus [34]. While quantitative virus outgrowth assays could not be performed here due to limited blood volumes, the newly described Intact Proviral DNA Assay (IPDA) [35, 36] could prove useful for pediatric studies. Interestingly, though, work from the Siliciano and Keele groups suggests that in nonhuman primates total cell-associated SIV DNA may more closely approximate the intact reservoir than in humans [35, 37]. A recent study by Garcia-Broncano *et al*. detailed novel molecular single-genome sequencing techniques allowing an in-depth analysis of the viral reservoir composition in HIV-1-infected infants using small blood volumes [38]. It is possible that, by using such techniques, we may have uncovered specific viral reservoir parameters impacted by TV+TLR7 and these methodologies should be incorporated into future studies. Nevertheless, ART interruption is still considered to be the most robust readout (and highest bar) to measure the outcome of a cure-directed intervention. Following analytical treatment interruption in this study, all infants rebounded with detectable viremia within 6 to 28 days and achieved viral set point with no detectable difference between experimental groups. These data suggest that although the infants demonstrate the capability to mount an immune response following vaccination that is detectable at ART interruption, this response was not sufficient to reduce viral reservoirs or set-point viremia following rebound.

Notably, although all vaccinated infants demonstrated an increase in the antiviral cellular immune response, the magnitude and breadth of the individual responses were variable between infants. When vaccinated infants were subdivided into strong and weak responders based on their SIV-specific T cell responses at the time of ATI measured by ELISPOT, cells triple positive for IFN-*γ*, TNF-*α*, and IL-2 quantified through ICS following MVA boost was only observed in strong responders. Through the comparison of strong and weak vaccine responders we have also identified a distinct immunological activation signature associated with the magnitude of vaccine response in our infants. Infants with a strong vaccine response show significantly higher HLA-DR expression on effector memory CD8^+^ T cells and a trend towards higher PD-1 expression in both central and effector memory CD8^+^ T cell subsets. Despite these described differences and consistent with the rest of our reported results, no difference in time to rebound or viral set point was observed between strong and weak vaccine responders. Although it is difficult to draw meaningful conclusions given the limited sample size, these results suggest that infants with a more activated memory CD8^+^ T cell repertoire may be more responsive to therapeutic vaccination strategies.

The results presented in this study are distinct from previously published data in adult RMs in which a similar vaccine regimen resulted in delayed viral rebound and reduced viral set point following ATI [18]. We note that the magnitude of IFN-*γ* response in infants after the final immunization was just over 50% of that observed in adults RMs in this prior work and the breadth of responses was also more restricted in infants compared to adults [18], perhaps accounting for some of the differences we observed in viral dynamics after ATI. Other experimental factors such as route of infection (oral vs. intrarectal), timing of ART initiation (4 weeks vs. 1 week post infection), and the use of different Ad vectors (Ad48 vs. Ad26) may also have influenced the different outcomes in infant vs. adult RMs. We hypothesize that the exponential increase in the size of the viral reservoir when ART is initiated at 4 weeks compared to 1 week after infection may have had the most significant impact on our results, although experimental evidence will be needed for confirmation. Additionally, distinct features of pediatric immunity during development or variance in composition of the viral reservoir between these age groups may be key [7, 19, 39–43]. Our findings highlight the importance of a pediatric model to evaluate HIV-1 cure interventions in this unique setting of immune development.

As this study was the first of its kind performed in infant RMs, there were several limitations. The small size of infant RMs and collection limitations restricted blood volumes and biopsy frequencies thereby reducing our capacity to perform extensive evaluative assays. Additionally, we are limited by small sample size when drawing comparisons between strong and weak vaccine responders within the TV+TLR7 RMs. However, we believe that studies such as the one presented here are critically important given the scarcity of HIV-1 cure and remission clinical trials in infants and children. Additionally, these findings provide key preclinical safety data on cure interventions in a pediatric HIV-1 animal model.

In conclusion, we have demonstrated that an Ad-prime, MVA-boost therapeutic immunization and repeated oral TLR-7 stimulation is safe and immunogenic in SIV-infected, ART-treated infant RMs. This finding represents a promising step forward in testing cure-directed interventions for pediatric HIV-1 using a model of breastmilk acquisition in which very early ART is not likely to be possible. Although we did not observe an effect on reservoir size or rebound dynamics, the immune response generated here may serve to eliminate infected cells if elicited contemporaneously with virus reactivation induced by an effective latency reversal agent. Future studies exploring therapeutic vaccination combined with latency reversal to clear virally infected cells may reveal the full potential of this cure approach.

## Materials and methods

### Ethics statement

This study was conducted in strict accordance with USDA regulations and the recommendations in the Guide for the Care and Use of Laboratory Animals of the National Institutes of Health, and were approved by the Emory University Institutional Animal Care and Use Committee (Protocol # YER-3000510-ELMNTS-A).

### Animals and SIV infection

Sixteen infant Indian RMs (*Macaca mulatta*), with exclusion of Mamu B*08 and B*17 positive animals, were enrolled in this study. The animals were born at the Yerkes National Primate Research Center (YNPRC) to dams housed in indoor/outdoor group housing. The infants were removed from the dams when they were approximately 2 weeks old and transferred to a nursery, where they were housed in social pairs with either full contact or protected contact for the duration of the study. The infants were fed in accordance with the YNPRC standard operating procedures (SOPs) for NHP feeding and had continual access to water. After being removed from the dam, infants were fed center approved milk replacer (Similac Advance, OptiGro Infant Formula with Iron and/or Similac Soy Isomil OptiGro Infant Formula with Iron; Abbott Nutrition, Columbus, OH) until 14 weeks of age. Infants were provided softened standard primate jumbo chow biscuits (Jumbo Monkey Diet 5037; Purina Mills, St. Louis, MO) and a portion of orange starting between 2–4 weeks of age. As animals aged additional enrichment of various fresh produce items were provided daily. Cages also contained additional sources of animal enrichment including objects such as perching and other manipulanda. Animal welfare was monitored daily. Appropriate procedures were performed to ensure that potential distress, pain, or discomfort was alleviated. The sedatives Ketamine (10 mg/kg) or Telazol (4 mg/kg) were used for blood draws and biopsies. Euthanasia of RMs, using Pentobarbital (100 mg/kg) under anesthesia, was performed only when deemed clinically necessary by veterinary medical staff and according to IACUC endpoint guidelines. The animals were orally infected at 4 to 5 weeks of age with two consecutive doses of 10^5^ TCID_50_ of SIV_mac251_. Four infants required multiple weekly 2-dose challenges prior to successful infection (range two to five challenges). Two of these RMs (one each allocated to the vaccine [RCc19] and control [RGc19] groups) were orally challenged with two consecutive doses of 10^5^ TCID_50_ SIV_mac239_ after three and four unsuccessful challenges with SIV_mac251_, respectively.

### Antiretroviral therapy

The sixteen RM infants were treated with a three-drug ART regimen initiated at 4 weeks post infection. The preformulation ART cocktail contained two reverse transcriptase inhibitors, 5.1 mg/kg Tenofovir Disoproxil Fumarate (TDF) and 40 mg/kg Emtricitabine (FTC), plus 2.5 mg/kg of the integrase inhibitor Dolutegravir (DTG). This ART cocktail was administered once daily at 1 mg/kg via the subcutaneous route.

### Vaccine regimen

Monkeys assigned to the TV+TLR7 group were primed by the intramuscular route with 3 x 10^10^ viral particles of Ad48 vectors [23] expressing SIV_smE543_ *gag-pol-env* at weeks 22 and 30 post infection and were boosted with 10^8^ plaque-forming units of MVA vectors [44] expressing SIV_smE543_ *gag-pol-env* at weeks 38 and 50 post infection. TV+TLR7 animals also received 10 administrations of 0.3 mg/kg GS-986 (Gilead Sciences) by oral gavage every two weeks from week 40–48 and 52–60 post infection.

### Sample collection and processing

EDTA-anticoagulated blood samples were collected regularly and used for a complete blood count, routine chemical analysis and immunostaining, with plasma separated by centrifugation within 1 h of phlebotomy. PBMCs were prepared by density gradient centrifugation. Lymph node and rectal biopsy tissue samples were collected at indicated timepoints (Fig 1). Lymph nodes were ground using a 70-μm cell strainer. Rectal biopsy mucosal mononuclear cells were isolated by digestion with collagenase and DNase I for 2 h at 37°C and then passed through a 70-μm cell strainer. The cell suspensions obtained were washed and immediately used for immunostaining or cryopreserved at -80°C until use.

### Immunophenotype by flow cytometry

Multicolor flow cytometric analysis was performed on whole blood or cell suspensions using predetermined optimal concentrations of the following fluorescently conjugated monoclonal antibodies (MAbs). For whole blood (WB) T cell analysis the following MAbs were used: CD3-allophycocyanin (APC)-Cy7 (clone SP34-2), CD95-phycoerythrin (PE)-Cy5 (clone DX2), Ki67-AF700 (clone B56), HLA-DR-peridinin chlorophyll protein (PerCP)-Cy5.5 (clone G46-6), CCR7-fluorescein isothiocyanate (FITC) (clone 150503), CCR5-APC (clone 3A9), and CD45-RA-PE-Cy7 (clone L45) from BD Biosciences; CD8-BV711 (clone RPA-T8), CD4-BV650 (clone OKT4), and PD-1-BV421 (clone EH12.2H7) from BioLegend; and CD28-ECD (clone CD28-2) from Beckman-Coulter. For WB activation analysis the following MAbs were used: CD3-allophycocyanin (APC)-Cy7 (clone SP34-2) and CD69-phycoerythrin (PE)-CF594 (clone FN50) from BD Biosciences; CD8-BV711 (clone RPA-T8), CD4-BV650 (clone OKT4), CD16-BV421 (clone 3G8), CD14-phycoerythrin (PE)-Cy7 (clone M5E2), and CD169-phycoerythrin (PE) (clone 7–239) from BioLegend; and CD38-allophycocyanin (APC) (clone OK10) from the NHP Reagent Resource. Flow cytometric acquisition and analysis of samples were performed on at least 100,000 events on an LSR II flow cytometer driven by the FACSDiva software package (BD Biosciences) or an AURORA flow cytometer driven by the SpectroFlo software package (Cytek). Analyses of the acquired data were performed using FlowJo version 10.0.4 software (TreeStar). For analysis, T cells were gated as live CD3^+^ cells and monocytes were gated as live CD3^-^CD4^int^ cells positive for either CD14 or CD16.

### IFN-*γ* ELISPOT

SIV-specific cellular immune responses were assessed by IFN-*γ* ELISPOT assays. Cryopreserved cells from selected timepoints pre- and post-immunization were thawed and rested overnight prior to assay. Test and control wells were performed with 200,000 cells per well in duplicate. Briefly, a 96-well MultiScreen-IP Filter Plate (Millipore) was activated with 70% EtOH for 1 min and washed twice with PBS. Plate was then incubated overnight at 4° with 5 μg/mL anti-human IFN-*γ* (MabTech). 200,000 PBMC were used per well and incubated with SIV_mac239_ peptide pools (NHP AIDS Reagent Program) at 10 μg/ml for 28 h at 37° in 5% CO_2_. SIV_mac239_ peptide pools were selected due to shared sequence identity of 97% with the challenge virus (SIV_mac251_) [45]. Due to limited cell availability, ELISPOTs were not performed using vaccine strain (SIV_smE543_) peptides; Gag, Pol, and Env from SIV_smE543_ share sequence identity with SIV_mac239_ of 91, 93, and 85%, respectively [46, 47]. As a positive control 5 μg/ml of concanavalin A (Millipore) was added to the cells, and a negative control of no peptide was also included on each plate. To estimate cellular breadth, cryopreserved cells from weeks 67 and 72 (weeks 3 and 8 post ATI) were utilized. This analysis was not possible after vaccination but before ATI due to limited cell availability. We note that the AUC of viremia during this time period was not different between TV+TLR7 and control groups (P = 0.46), permitting some inferences about the vaccine strategy to be considered. 10-mer peptides were divided into subpools of 20 peptides per subpool spanning the entire Gag, Pol, or Env SIV_mac239_ viral protein. All tests were performed in duplicate. Plates were scanned using an automated ELISPOT counter (CTL, Cellular Technologies), and verified through manual counting. Background (the mean of wells without peptide stimulation) levels were subtracted from each well on the plate. A response was considered positive if the mean number of spot-forming cells (SFC) from duplicate sample wells exceeded background plus 2 standard deviations. Assay results are shown as SFC per 1 x 10^6^ cells. Responses of <50 SFC per 1 x 10^6^ cells were not considered positive.

### Intracellular cytokine staining (ICS)

ICS was performed using fresh or cryopreserved PBMCs. Cryopreserved PBMCs were rested over night after thaw prior to assay. Briefly, 1–2 x 10^6^ cells were incubated in the presence of Brefeldin A (Sigma Aldrich), BD Golgi Stop (BD BioSciences), and SIV_mac239_ Gag peptide pool (NHP AIDS Reagent Program) for 6 hours at 37°. PMA/Ionomycin (Sigma Aldrich) was used as a positive control and no peptide stimulation was used as the negative control. After incubation, the cells were washed twice with PBS and stained for 20 minutes at 37° with LIVE/DEAD then for 30 minutes at RT with predetermined optimal concentrations of the following fluorescently conjugated MAbs: CD3-APC-Cy7 (clone SP34-2), CD4-BV421 (clone OKT4), CD8-PE-CF594 (clone RPA-T8), and CD95-PE-Cy5 (Clone DX2). After the incubation, cells were fixed and permeabilized and then incubated with the following MAbs: IL-17A-AF488 (clone eBio64DEC17), IFN-*γ*-PE (clone B27), TNF*α*-AF700 (clone Mab11), IL-2-BV605 (clone MQ1-17H12), IL-22-APC (clone IL22JOP). Flow cytometric acquisition and analysis of samples were performed on at least 100,000 events on an LSR II flow cytometer driven by the FACSDiva software package (BD Biosciences). Analyses of the acquired data were performed using FlowJo version 10.0.4 software (TreeStar) and simplified presentation of incredibly complex evaluations (SPICE, v.6.0) software.

### ELISA and Binding Antibody Multiplex Assay (BAMA)

Enzyme-linked immunosorbent assays were conducted to assess IgG binding to SIV antigens. High-binding 384-well plates (Corning Life Sciences) were coated overnight at 4°C at 2 ug/ml with gp120 from SIV_mac251_ (Creative Biolabs), SIV_smE543_, or gp140 from SIV_smE543_ (Immune Technology Corps, New York, NY). Plates were blocked with assay diluent (PBS containing 4% whey protein, 15% goat serum, 0.5% Tween 20) for 1 h at 20°C. Plasma dilutions were added to the plate and incubated for 1 h at 20°C. IgG was detected by a horse radish peroxidase (HRP)-conjugated mouse anti-monkey IgG polyclonal antibody (Southern Biotech). ELISA plates were developed with SureBlue Reserve TMB substrate and stop solution (KPL). Plates were read immediately after addition of stop solution at 450 nm, 0.1 s/well on a SpectraMax Plus 384 microplate reader (Molecular Devices). Area under the curve was calculated using the GraphPad Prism Software. An anti-SIV_mac251_ polyclonal IgG made from pooled sera from six SIV_mac251_ challenged RMs (AIDS Reagent Program) was used as positive control. Binding Antibody Multiplex Assays (BAMAs) were performed as previously described [48]. Briefly, SIV antigens were first coupled to carboxylated fluorescent beads (Bio-Rad Laboratories, Inc.). The antigen panel included SIV_mac239_ gp120, Nef (Immune Technology Corps), and p27 (AIDS Reagent Program). The coupled beads were incubated with diluted plasma (1:50 or 1:2500) for 30 min at 20°C and then IgG binding was detected with streptavidin-conjugated mouse anti-monkey IgG at 0.5 μg/ml. Beads were washed and read on a Bio-Plex 200 instrument (Bio-Rad Laboratories, Inc.). IgG levels were expressed as mean fluorescent intensity (MFI) and all mean fluorescent intensity (MFI) values were blank bead and well subtracted. Consistency between assays was ensured by tracking the maximum MFI and AUC of the positive control (anti-SIV_mac251_ polyclonal IgG) by Levy-Jennings charts.

### Plasma RNA and cell-associated DNA viral quantification

Plasma viral quantification was performed as described previously [17]. Frozen cell pellet was lysed with proteinase K (100 μg/ml in 10 mM Tris-HCl pH 8) for 1 h at 56°C. Quantification of SIV_mac_ *gag* DNA was performed by quantitative PCR using the 5’ nuclease (TaqMan) assay with an ABI7500 system (PerkinElmer Life Sciences). The sequence of the forward primer for SIV_mac_ *gag* was 5′-GCAGAGGAGGAAATTACCCAGTAC-3′, the reverse primer sequence was 5′-CAATTTTACCCAGGCATTTAATGTT-3′, and the probe sequence was 5′-6-carboxyfluorescein (FAM)-TGTCCACCTGCCATTAAGCCCGA-6-carboxytetramethylrhodamine (TAMRA)-3′. 7.5 μL of cell lysate were mixed in a 50 μL reaction containing 1x Platinum Buffer, 3.5 mM MgCl_2_, 0.2 mM dNTP, primers 200 nM, probe 150 nM, and 2 U Platinum Taq. For cell number quantification, quantitative PCR was performed simultaneously for monkey albumin gene copy number. The sequence of the forward primer for albumin was 5’-TGCATGAGAAAACGCCAGTAA-3’; the reverse primer sequence was 5’- ATGGTCGCCTGTTCACCAA-3’ and the probe sequence was 5’- AGAAAGTCACCAAATGCTGCACGGAATC-3’ [49]. The reactions were performed on a 7500 real-time PCR system (Applied Biosystems) with the following thermal program: 5 min at 95°C, followed by 40 cycles of denaturation at 95°C for 15 s and annealing at 60°C for 1 min.

### Statistical analyses

Statistical analyses were performed using GraphPad Prism Software (v.7 or v.8). *P* ≤ 0.05 was considered statistically significant. To test the statistical significance observed in magnitude of T cell response in Fig 2B and humoral response Fig 5 within groups, the non-parametric Friedman test with Dunn’s correction for multiple comparisons was used. To compare memory CD4^+^ and CD8^+^ polyfunctionality in Fig 3 and SIV DNA levels in Fig 7A and 7B a two-sided Wilcoxon rank-sum test was used. To compare the magnitude of T cell responses to viral subpools in Fig 4A and to compare the frequency of CD38 and CD69 expression on CD4^+^ and CD8^+^ T cells and CD169 expression on monocyte subsets in Figs 6 and S7 a Wilcoxon matched-pairs signed rank test was performed. A two-sided Spearman Rank correlation test was used to determine statistical significance of associations in Figs 7C and 9D–9F. In Fig 8A survival curves were compared through a Log-rank (Mantel-Cox) test. To compare differences between groups in pre-ART VL and CD4^+^ T cell frequency in S2B and S2C Fig, positive subpools in Figs 4B and S6B, humoral response between groups in Fig 5, VL set point in Fig 8C, magnitude of the anti-SIV ELISPOT response in Fig 9A, AUC between groups in Fig 9C, and rebound kinetics in Fig 9I a two-sided Mann-Whitney was used.

## Supporting information

S1 Fig On ART plasma viral loads of TV+TLR7 and control RMs. Longitudinal analysis of plasma SIV RNA levels in (A) TV+TLR7 and (B) control RMs. The shaded area represents the period of ART treatment. The colored dashed lines represent therapeutic intervention in TV+TLR7 RMs, Ad48 is in green, MVA is in blue, and GS-986 in orange. The horizontal dashed line represents the limit of detection of the assay.
(TIF)

S2 Fig Comparison of challenge variables on acute viral kinetics. (A) Longitudinal analysis of plasma SIV RNA levels by required number of challenges prior to successful infection. The shaded area represents the period of ART treatment. RMs that required one challenge, two challenges, three challenges, and four challenges are represented by black, green, purple, and orange lines, respectively. Pre-ART viral kinetics were not influenced by (B) required number of challenges or (C) challenge virus. Groups were compared using a two-sided Mann-Whitney test (P < 0.05 was considered significant). Bars represent mean ± SD.
(TIF)

S3 Fig Oral GS-986 is tolerable, safe, and induces anticipated immune stimulation at 0.1 and 0.3 mg/kg in SIV-infected, ART-treated infant RMs. (A) Longitudinal assessment of body weight, complete blood counts, and serum chemistries. The shaded areas represent the period of ART treatment. The dotted lines represent the normal range for each parameter. WBC, white blood cells; HGB, hemoglobin; BUN, blood urea nitrogen; ALT, alanine aminotransferase; GGT, gamma-glutamyltransferase. (B) Frequency of CD169^+^CD14^+^ monocytes before, 1 day after, and 7 days after oral GS-986. Representative staining for CD169 expression within CD14^+^ monocytes is shown on the right.
(TIF)

S4 Fig Safety data in ART-treated SIV-infected RM infants. (A) Longitudinal assessment of complete blood counts (WBC, white blood cells; HGB, hemoglobin) (B) body weight, and (C) serum chemistries (BUN, blood urea nitrogen; ALT, alanine aminotransferase; GGT, gamma-glutamyltransferase). The shaded areas represent the period of ART treatment. The dotted lines represent the normal range for each parameter.
(TIF)

S5 Fig Individual IFN-*γ* ELISPOT responses of control RMs. IFN-*γ* ELISPOT responses to Gag, Pol, and Env peptide pools from SIV_mac239_ were measured at 22, 34 52, and 62 weeks post infection (p.i.). SFC = spot forming cells.
(TIF)

S6 Fig Cellular breadth to individual peptide pools following analytical treatment interruption (ATI) measured by ELISPOT. (A) Individual IFN-*γ* ELISPOT responses to 10-mer peptide subpools spanning the Gag, Pol, and Env proteins from SIV_mac239_ following ATI in TV+TLR7 and control RMs. Bars represent median ± quartiles and the gray shading bordered by the horizontal dashed line represents the limit of detection. (B) Cellular immune breadth in TV+TLR7 RMs and control RMs as measured by positive subpools of 10 peptides spanning the SIV_mac239_ Gag, Pol, and Env proteins following ATI. Black bar represents median. Groups were compared using a two-sided Mann-Whitney test (P < 0.05 was considered significant).
(TIF)

S7 Fig Detailed immunological response to repeated oral GS-986 administration in SIV-infected, ART-treated infant RMs. Frequency of (A) CD38^+^ and (B) CD69^+^ peripheral CD4^+^ and CD8^+^ T cells immediately prior to, 1 day post, and 14 days post oral GS-986 doses 1, 5, and 10 in TV+TLR7 or equivalent sample day in control RMs, shown by individual animal. (C) Frequency of CD169^+^ classical, intermediate, and nonclassical monocytes in TV+TLR7 and control RMs. Dose number is indicated by color. Bars represent mean ± SEM. Statistical analysis was performed using a Wilcoxon matched-pairs signed rank test.
(TIF)

S8 Fig Post analytical treatment interruption (ATI) plasma viral loads of TV+TLR7 and control RMs. Longitudinal analysis of plasma SIV RNA levels in (A) TV+TLR7 and (B) control RMs following ATI. The horizontal dashed line represents the limit of detection of the assay.
(TIF)

S9 Fig Impact of therapeutic vaccination and oral TLR-7 stimulation on reservoir between good and poor vaccine responders within TV+TLR7 infant RMs. Comparison of frequency of estimated SIV DNA levels and log reduction from week 12 to week 62 in (A) peripheral and (B) LN CD4^+^ T cell SIV DNA in strong and weak responders as determined by PCR.
(TIF)
