# Supplementary figures and images for: Therapeutic vaccination of SIV-infected, ART-treated infant rhesus macaques using Ad48/MVA in combination with TLR-7 stimulation
Source: PLoS Pathog. 2020 Oct 26;16(10):e1008954. doi: 10.1371/journal.ppat.1008954 (PMC7644092; doi:10.1371/journal.ppat.1008954)

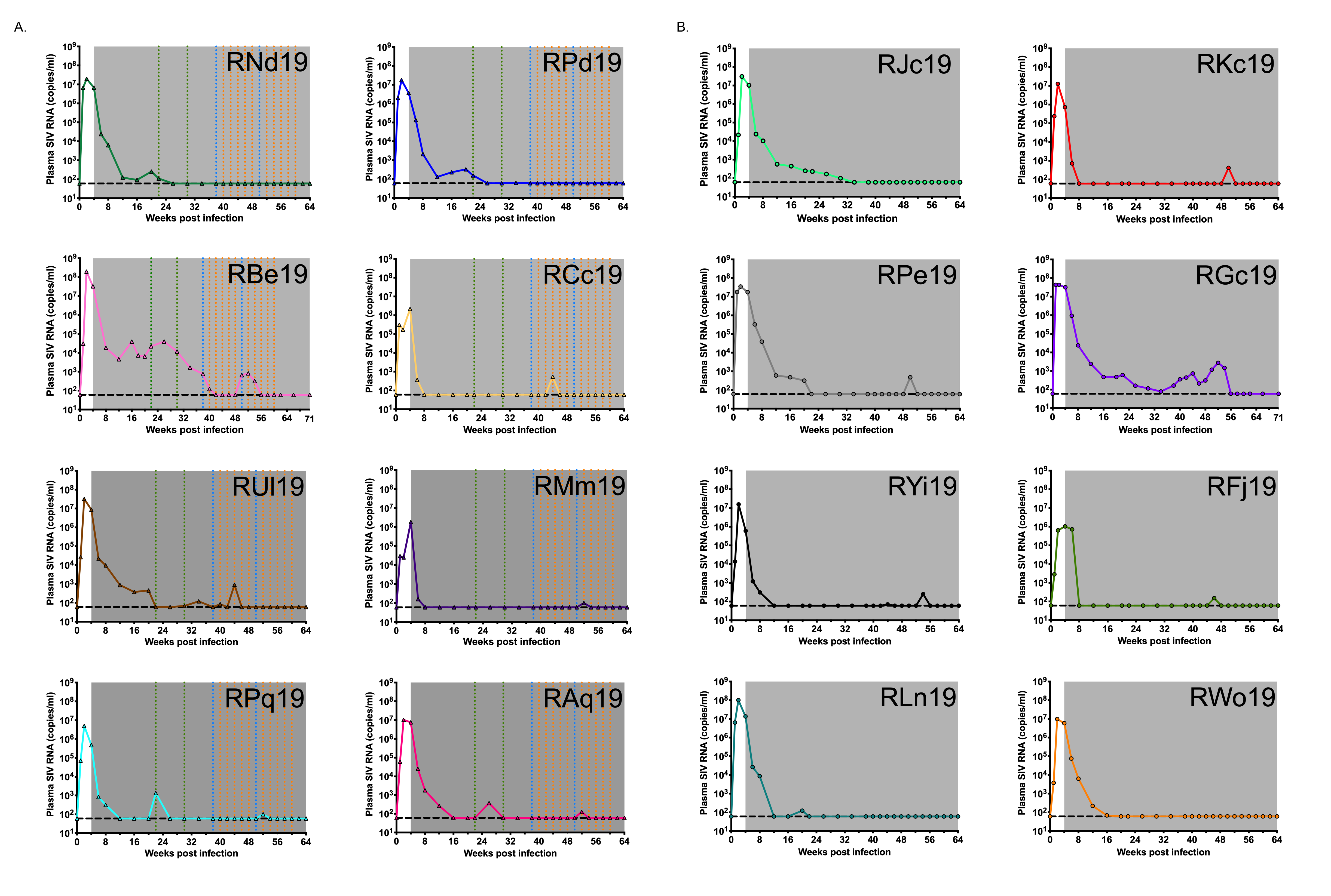

Supplement: S1 Fig — Longitudinal analysis of plasma SIV RNA levels in (A) TV+TLR7 and (B) control RMs. The shaded area represents the period of ART treatment. The colored dashed lines represent therapeutic intervention in TV+TLR7 RMs, Ad48 is in green, MVA is in blue, and GS-986 in orange. The horizontal dashed line represents the limit of detection of the assay. (TIF) [file ppat.1008954.s001.tif]

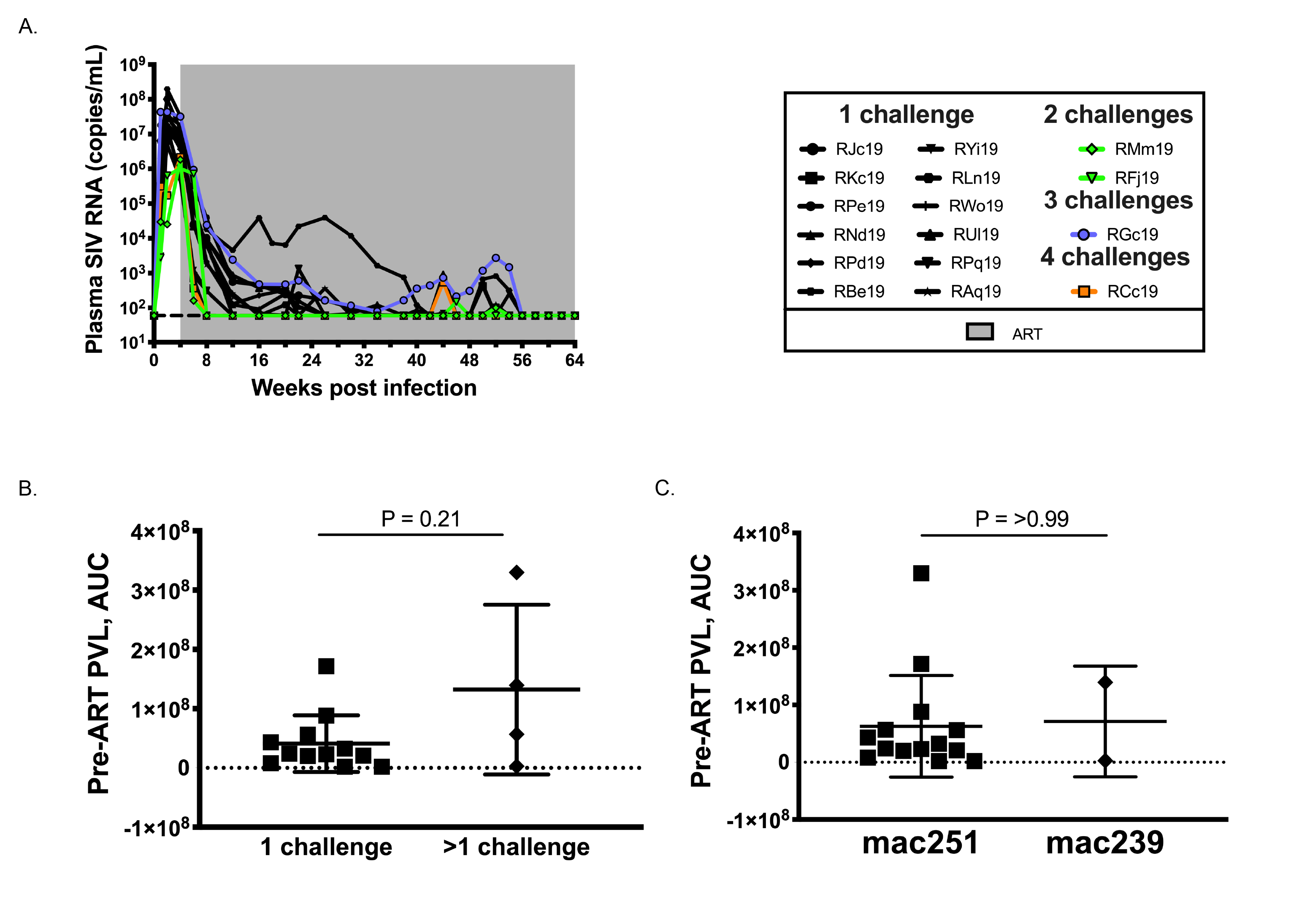

Supplement: S2 Fig — (A) Longitudinal analysis of plasma SIV RNA levels by required number of challenges prior to successful infection. The shaded area represents the period of ART treatment. RMs that required one challenge, two challenges, three challenges, and four challenges are represented by black, green, purple, and orange lines, respectively. Pre-ART viral kinetics were not influenced by (B) required number of challenges or (C) challenge virus. Groups were compared using a two-sided Mann-Whitney test (P < 0.05 was considered significant). Bars represent mean ± SD. (TIF) [file ppat.1008954.s002.tif]

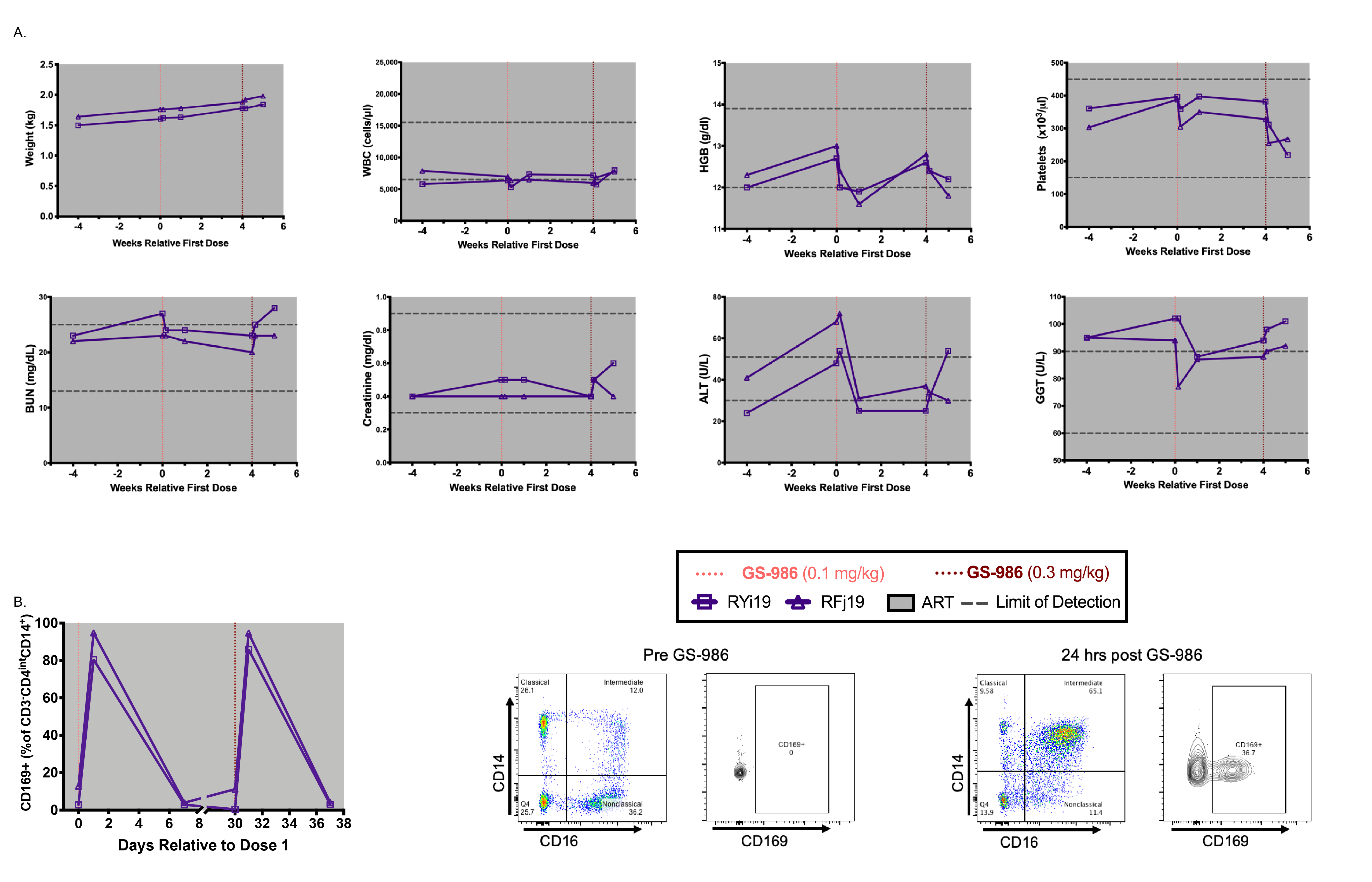

Supplement: S3 Fig — (A) Longitudinal assessment of body weight, complete blood counts, and serum chemistries. The shaded areas represent the period of ART treatment. The dotted lines represent the normal range for each parameter. WBC, white blood cells; HGB, hemoglobin; BUN, blood urea nitrogen; ALT, alanine aminotransferase; GGT, gamma-glutamyltransferase. (B) Frequency of CD169+CD14+ monocytes before, 1 day after, and 7 days after oral GS-986. Representative staining for CD169 expression within CD14+ monocytes is shown on the right. (TIF) [file ppat.1008954.s003.tif]

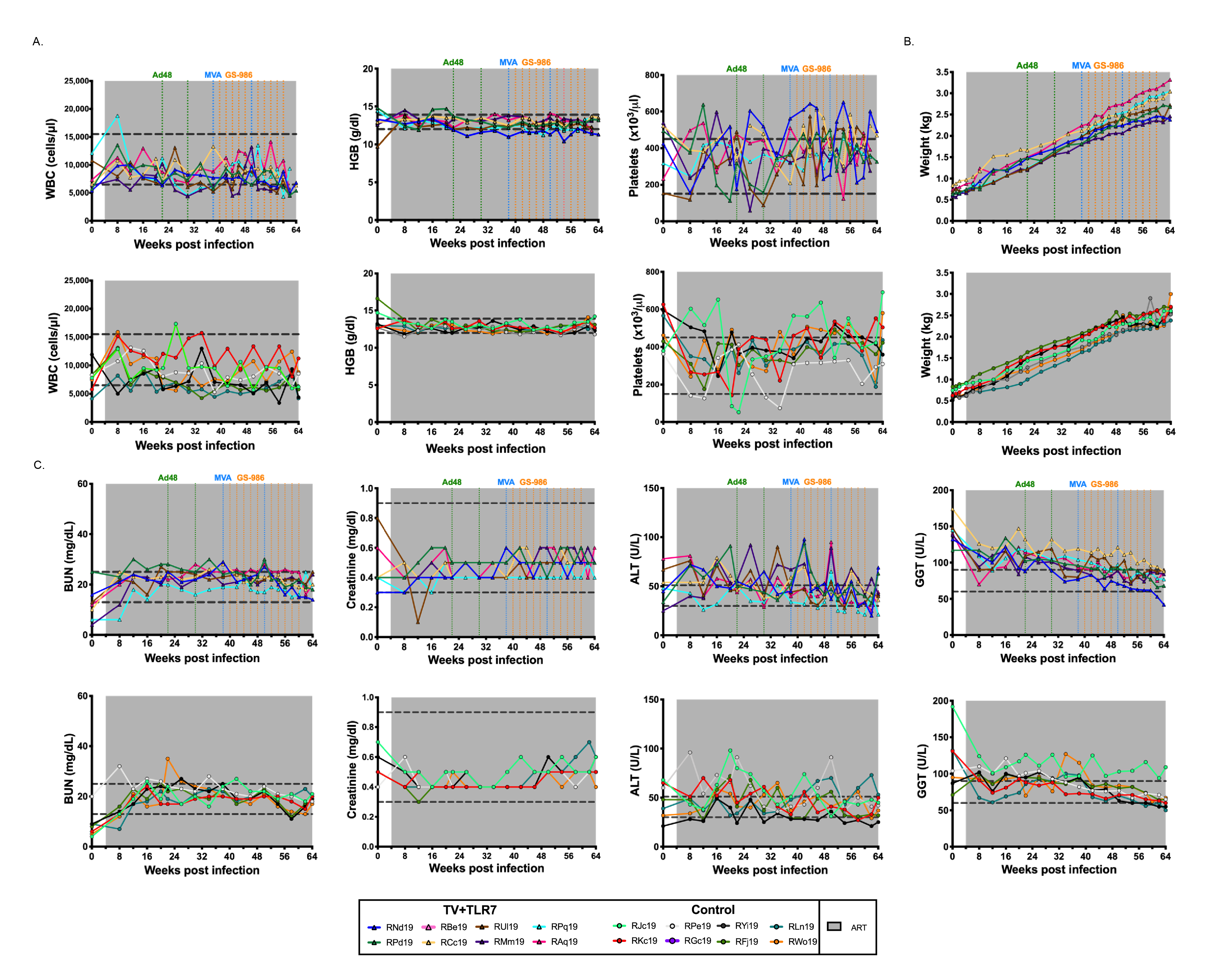

Supplement: S4 Fig — (A) Longitudinal assessment of complete blood counts (WBC, white blood cells; HGB, hemoglobin) (B) body weight, and (C) serum chemistries (BUN, blood urea nitrogen; ALT, alanine aminotransferase; GGT, gamma-glutamyltransferase). The shaded areas represent the period of ART treatment. The dotted lines represent the normal range for each parameter. (TIF) [file ppat.1008954.s004.tif]

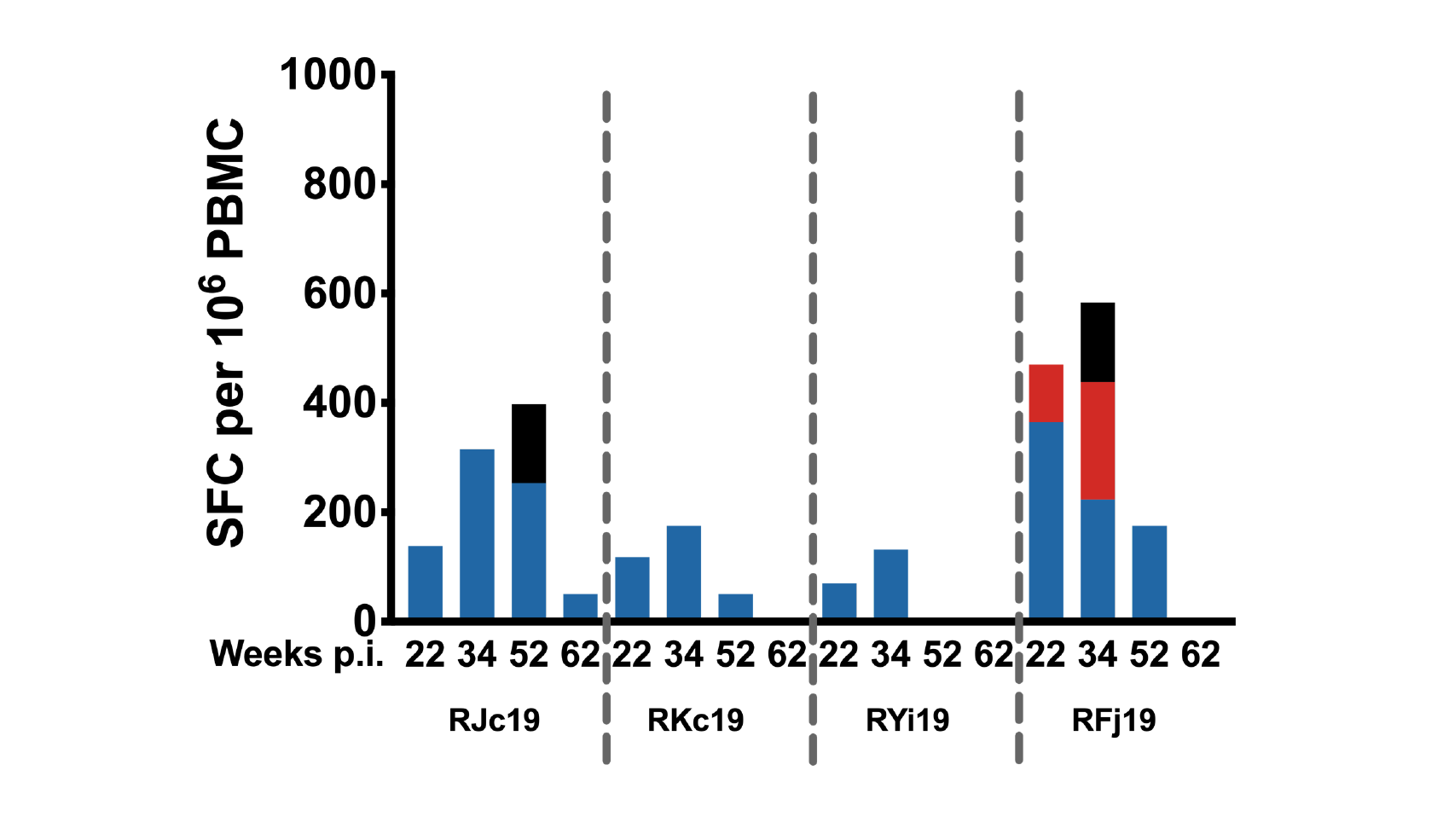

Supplement: S5 Fig — IFN-γ ELISPOT responses to Gag, Pol, and Env peptide pools from SIVmac239 were measured at 22, 34 52, and 62 weeks post infection (p.i.). SFC = spot forming cells. (TIF) [file ppat.1008954.s005.tif]

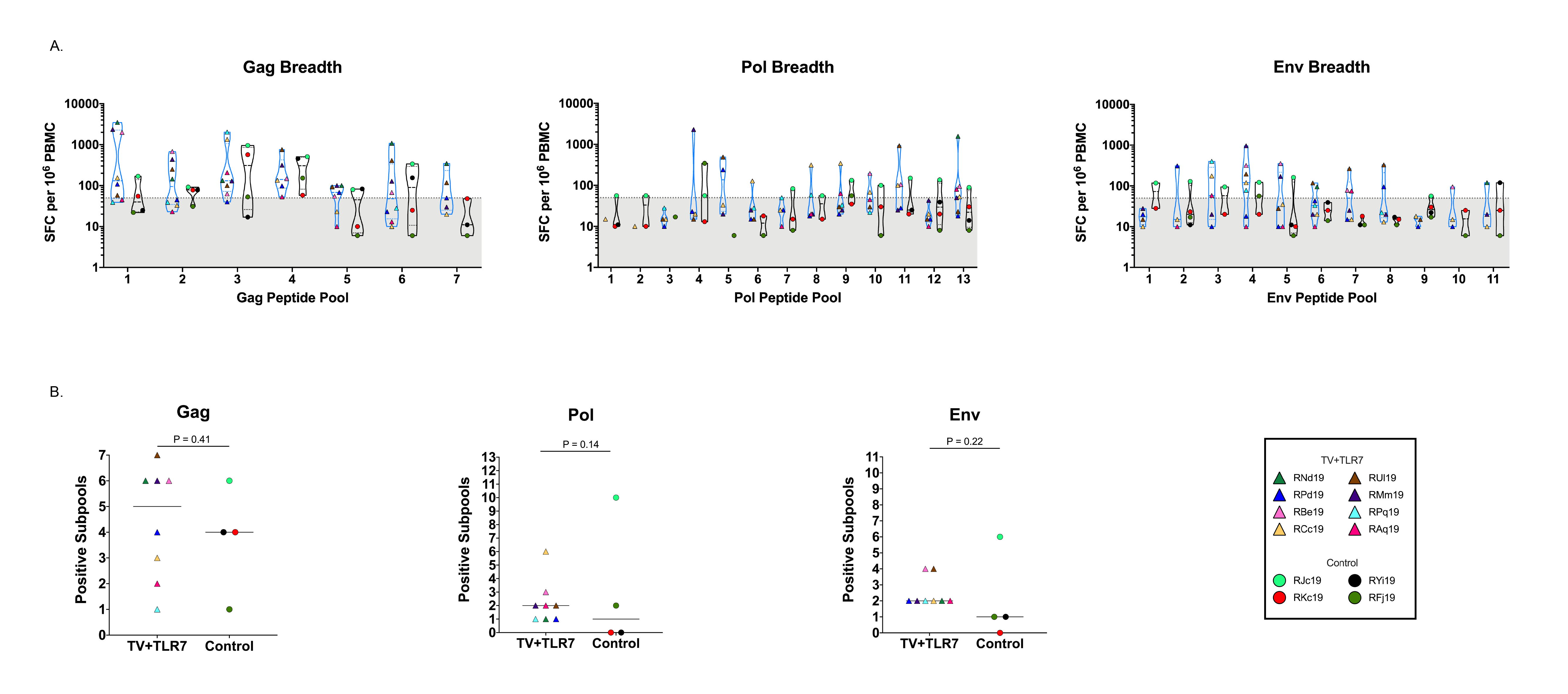

Supplement: S6 Fig — (A) Individual IFN-γ ELISPOT responses to 10-mer peptide subpools spanning the Gag, Pol, and Env proteins from SIVmac239 following ATI in TV+TLR7 and control RMs. Bars represent median ± quartiles and the gray shading bordered by the horizontal dashed line represents the limit of detection. (B) Cellular immune breadth in TV+TLR7 RMs and control RMs as measured by positive subpools of 10 peptides spanning the SIVmac239 Gag, Pol, and Env proteins following ATI. Black bar represents median. Groups were compared using a two-sided Mann-Whitney test (P < 0.05 was considered significant). (TIF) [file ppat.1008954.s006.tif]

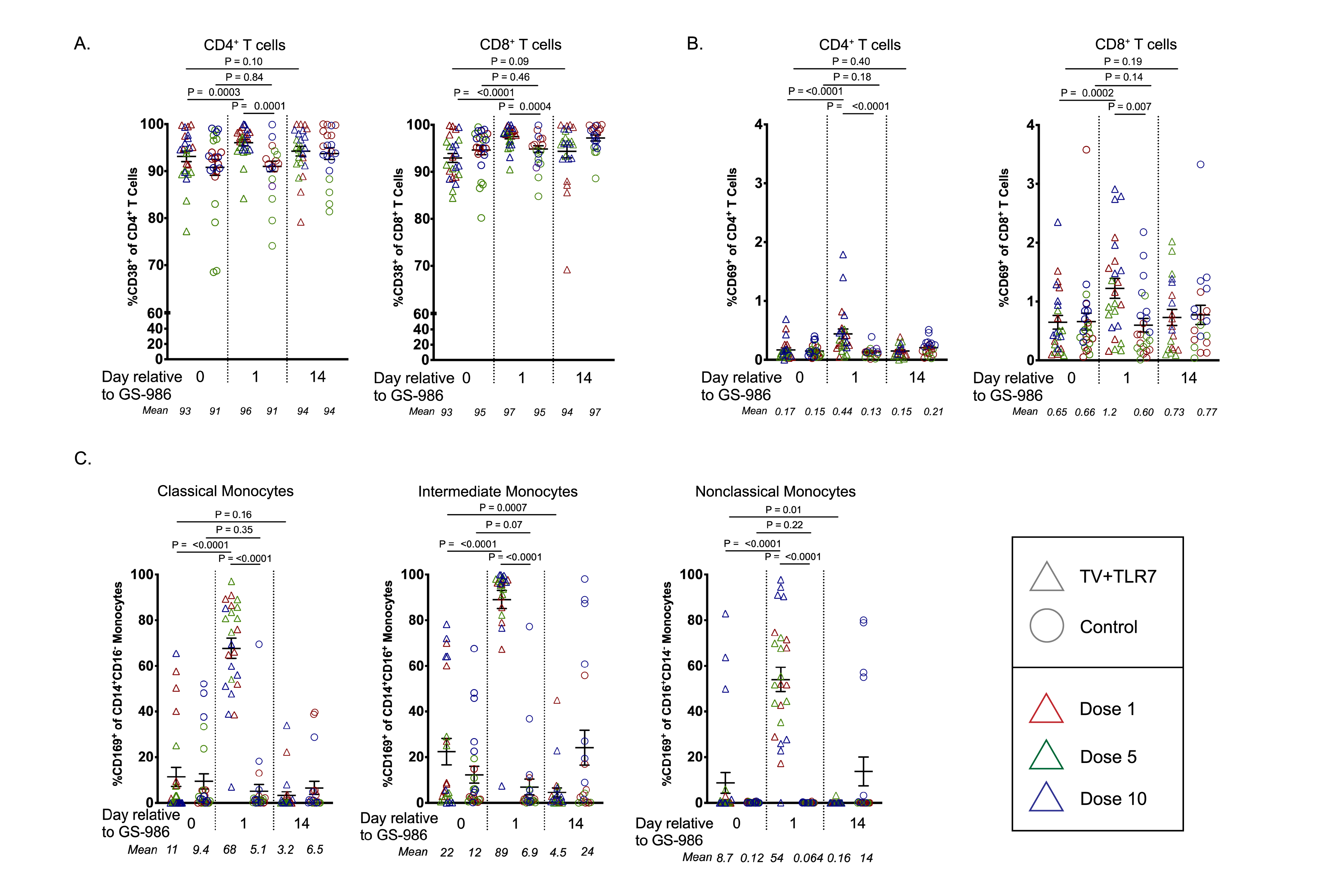

Supplement: S7 Fig — Frequency of (A) CD38+ and (B) CD69+ peripheral CD4+ and CD8+ T cells immediately prior to, 1 day post, and 14 days post oral GS-986 doses 1, 5, and 10 in TV+TLR7 or equivalent sample day in control RMs, shown by individual animal. (C) Frequency of CD169+ classical, intermediate, and nonclassical monocytes in TV+TLR7 and control RMs. Dose number is indicated by color. Bars represent mean ± SEM. Statistical analysis was performed using a Wilcoxon matched-pairs signed rank test. (TIF) [file ppat.1008954.s007.tif]

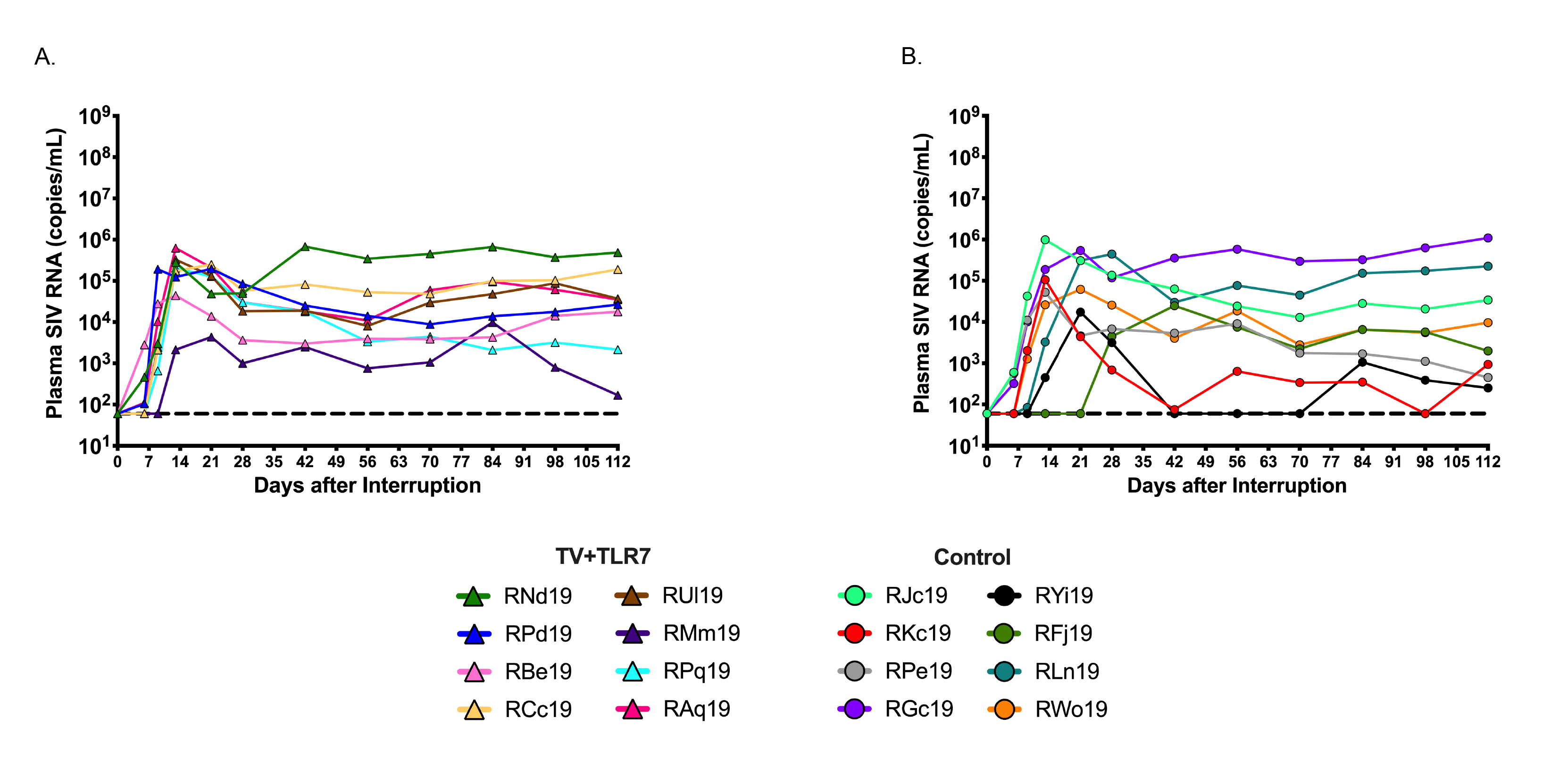

Supplement: S8 Fig — Longitudinal analysis of plasma SIV RNA levels in (A) TV+TLR7 and (B) control RMs following ATI. The horizontal dashed line represents the limit of detection of the assay. (TIF) [file ppat.1008954.s008.tif]

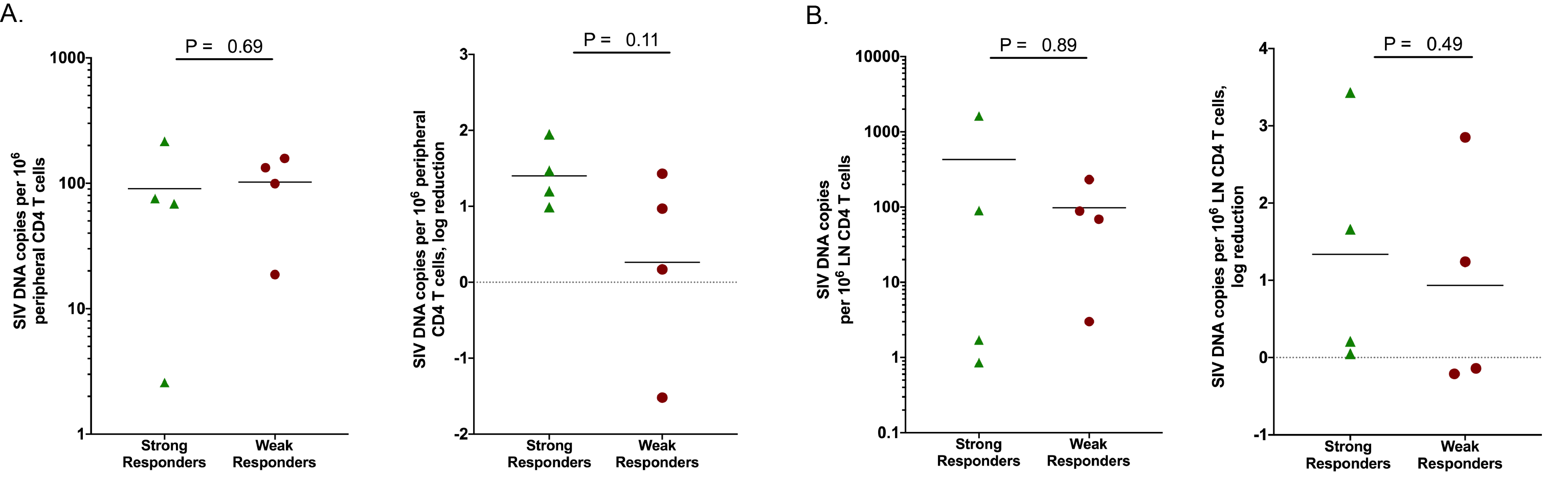

Supplement: S9 Fig — Comparison of frequency of estimated SIV DNA levels and log reduction from week 12 to week 62 in (A) peripheral and (B) LN CD4+ T cell SIV DNA in strong and weak responders as determined by PCR. (TIF) [file ppat.1008954.s009.tif]
